# Blocking the WNT/β-catenin pathway in cancer treatment:pharmacological targets and drug therapeutic potential

**DOI:** 10.1016/j.heliyon.2024.e35989

**Published:** 2024-08-12

**Authors:** Xi Zhao, Yunong Ma, Jiayang Luo, Kexin Xu, Peilin Tian, Cuixia Lu, Jiaxing Song

**Affiliations:** a Medical Scientific Research Center, Life Sciences Institute, Guangxi Medical University, Nanning, 530021, China; b China Medical College of Guangxi University, Guangxi University, Nanning, 530004, China

**Keywords:** WNT/β-catenin, Drug target, Drug discovery, Medicinal research, Drug development

## Abstract

The WNT/β-catenin signaling pathway plays crucial roles in tumorigenesis and relapse, metastasis, drug resistance, and tumor stemness maintenance. In most tumors, the WNT/β-catenin signaling pathway is often aberrantly activated. The therapeutic usefulness of inhibition of WNT/β-catenin signaling has been reported to improve the efficiency of different cancer treatments and this inhibition of signaling has been carried out using different methods including pharmacological agents, short interfering RNA (siRNA), and antibodies. Here, we review the WNT-inhibitory effects of some FDA-approved drugs and natural products in cancer treatment and focus on recent progress of the WNT signaling inhibitors in improving the efficiency of chemotherapy, immunotherapy, gene therapy, and physical therapy. We also classified these FDA-approved drugs and natural products according to their structure and physicochemical properties, and introduced briefly their potential mechanisms of inhibiting the WNT signaling pathway. The review provides a comprehensive understanding of inhibitors of WNT/β-catenin pathway in various cancer therapeutics. This will benefit novel WNT inhibitor development and optimal clinical use of WNT signaling-related drugs in synergistic cancer therapy.

## Graphical abstract

**Figure:**
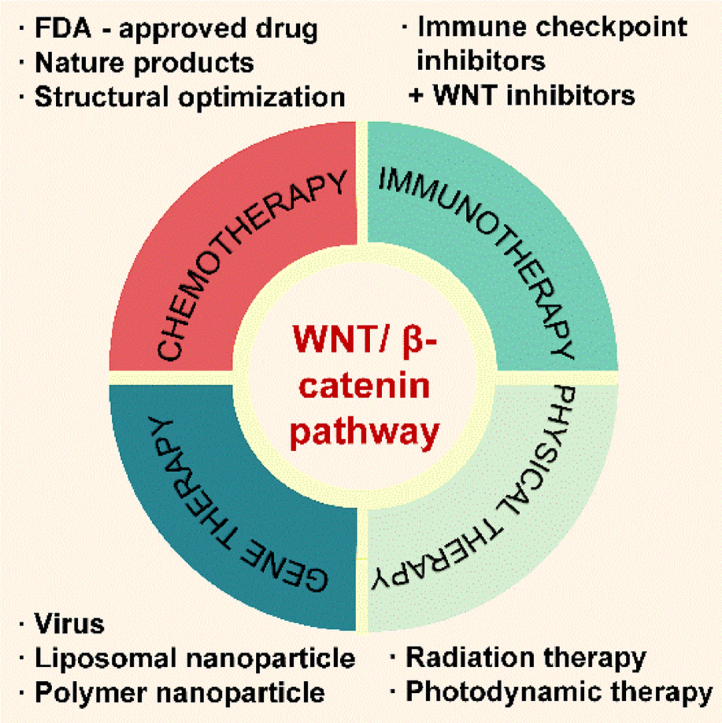

## 1 Introduction

The WNT/β-catenin signaling pathway plays an important role in organ formation, stem cell renewal, differentiation, and tissue maintenance. Abnormal activation of the WNT/β-catenin signaling pathway is closely associated with the initiation and progression of various types of cancer, such as breast cancer [1,2], colorectal cancer (CRC) [3,4], melanoma [5,6], prostate cancer [7,8], lung cancer [9,10], and other types of tumors [[11], [12], [13], [14]]. Activation of the pathway elevates transcription of WNT/β-catenin downstream target genes which are involved in tumor development, relapse, and metastasis [15]. Aberrant activation of the WNT signaling also makes cancer resistant to multiple therapeutic approaches by maintaining cancer stem cells (CSCs) populations, enhancing DNA damage repair, promoting transcriptional plasticity, and facilitating immune evasion [16]. Increasing data have demonstrated that manipulating the WNT signaling pathway could extremely improve the tumoricidal efficacy of cancer modalities such as chemotherapy, immunotherapy, gene therapy, and physical therapy. Therefore, it is essential to focus on the control of the WNT pathway in cancer treatment.

Herein, we provide an overview of the application of targeting the WNT pathway in chemotherapy, immunotherapy, gene therapy, and physical therapy, review the effect of controlling the WNT/β-catenin pathway for improving cancer treatment outcomes in each cancer modality, and stress the importance of regulating the WNT/β-catenin pathway in combination with various therapies to increase efficacy.

## 2 Brief intro of WNT/β-catenin

The name ‘WNT’ is a portmanteau created from the names Integration 1 (*Int-1*) and Wingless (*Wg*). *Int-1* is a proto-oncogene originated from mice [17], and subsequent studies have identified significant similarities between the *Int-1* gene and the *Wg* gene in Drosophila. Thus, the *Int-1* gene was renamed “*WNT*' [18]. In mammals, the WNT family consists of 19 known WNT members that are highly conserved in many species and function as ligands for activating cellular signal transduction. With progressive research, WNT signaling has emerged as a fundamental growth control pathway, including embryonic development, cell cycle regulation, inflammation, and cancer [19].

**Fig. 1:**
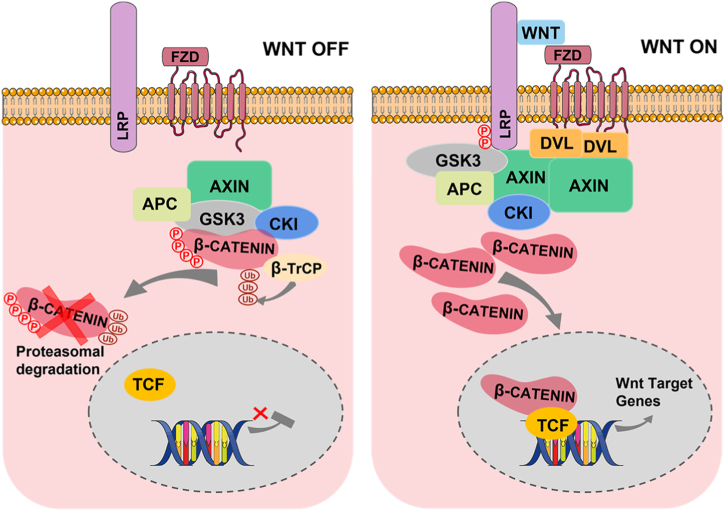
An overview of the WNT/β-catenin pathway. In the absence of WNT ligands, free β-catenin in the cytoplasm is bound by the β-catenin destruction complex consisting of APC, CK1, GSK-3β and AXIN. Phosphorylation of β-catenin leads to its ubiquitylation and subsequent proteasomal degradation. When WNT ligands are present, WNT binds to the FZD receptors and the co-receptor LRP5 or LRP6 on the cell membrane surface, induces phosphorylation of LRP5/6, which recruits DVL and AXIN, allowing the destruction complex to be disrupted, thus preventing degradation of β-catenin and leading to β-catenin accumulation in the cytoplasm and translocation into the nucleus. In the nucleus, β-catenin interacts with TCF/LEF transcription factors to activate the transcription of WNT target genes.

The WNT signaling pathway can be subdivided into two categories: canonical WNT signaling pathways and non-canonical signaling pathways. Canonical WNT signaling is also known as “WNT/β-catenin signaling”, which is activated mainly by regulating the accumulation of β-catenin in the cytoplasm. β-catenin is a core transcriptional co-activator of canonical WNT signaling and is tightly controlled by the β-catenin destruction complex consisting of adenomatous polyposis coli (APC), casein kinase 1 (CK1), glycogen synthase kinase 3β (GSK-3β) and axis inhibition protein (AXIN) [19,20]. In the absence of WNT ligands, free β-catenin in the cytoplasm is bound by the cytoplasmic destruction complexes, and the N-terminal of β-catenin is sequentially phosphorylated at residue S45 by CK1 and then at Ser33, Ser37, Thr41 by GSK-3β, which facilitates β-catenin to be recognized and ubiquitylated by the E3 ubiquitin ligase β-TrCP and finally results in β-catenin degradation via the proteasome pathway. When WNT ligands are present, WNT binds to the Frizzled (FZD) receptors on the cell membrane surface, as well as to the co-receptor low-density lipoprotein receptor-related protein 5 (LRP5) or LRP6, which recruits Dishevelled (DVL) and AXIN, induces phosphorylation of LRP5/6, allowing the degradation complex to be disrupted, thus preventing degradation of β-catenin and leading to accumulation of β-catenin in the cytoplasm. Afterward, cytoplasmic β-catenin undergoes nuclear translocation via an as-yet-undiscovered mechanism and interacts with the transcription factor TCF/LEF to initiate the transcriptional activation of downstream target genes (Fig. 1) [21,22]. In addition to binding with transcription factors TCF/LEF, β-catenin can also interact with other transcriptional factors like FOXO to regulate gene transcription [23].

In many cancers, there are frequent mutations in some members of the WNT/β-catenin signaling. For example, in CRC, APC mutations reach up to 80 % [24] and there are 48 % mutations in β-catenin in CRC lacking APC mutations [25]. However, APC or β-catenin mutations in breast cancer are very rare, only 6 % [26]. Conversely, the WNT receptor Frizzled 6 (FZD6) is frequently ampliﬁed (19.11 %) and overexpressed (18.71 %) in triple-negative breast cancer (TNBC) [27]. In hepatocellular carcinoma (HCC), activating mutations in β-catenin (*CTNNB1*) occurs by 30–44 % [28,29]. Of course, other WNT pathway-related genes like AXIN1 and AXIN2 mutation were detected in a smaller percentage [28]. Notably, most of the mutations lead to cause abnormal accumulation of β-catenin, especially mutation of β-catenin phosphorylation sites, which are sufﬁcient to activate WNT signaling.

Given the association between abnormal activation of WNT/β-catenin signaling and the pathogenesis of multiple cancers, and the fact that many cancers have mutations associated with the WNT pathway, this pathway has become an important target for cancer drug discovery. Despite advances in the development of biologics such as Vantictumab (human anti-FZD antibody) and Ipafricept (a recombinant fusion protein that competes to bind the WNT ligand), small-molecule drugs are currently the most promising therapeutic approaches for targeting the WNT pathway.

## 3 Chemotherapy

Chemotherapy can be traced back to the early 1900s when Paul Ehrlich discovered that chemotherapy drugs could treat cancer, and since then chemotherapy research on different cancers has begun [30]. Currently, chemotherapy is the most basic cancer treatment, and there are several chemotherapeutic agents used in cancer treatment, including platinum-based drugs [31], paclitaxel [32], oncology antibiotics [33], and antimetabolites [34], etc. However, the long-term application of chemotherapeutic agents in cancer treatment results in the development of drug resistance of cancer cells, which is closely related to the activation of the WNT/β-catenin signaling pathway. For example, 5-fluorouracil (5-FU), an anti-metabolic chemotherapeutic agent, can effectively inhibit tumor growth in the early stage of treatment. Owing to the activation of the WNT/β-catenin pathway and the enrichment of tumor stem cells in CRC, the tumor recurs after 5-FU treatment, which seriously influences the therapeutic effect of chemotherapy [35]. Several studies have also pointed out that WNT signaling activation leads to drug resistance of cancer cells to chemotherapeutic agents including platinum and paclitaxel [[36], [37], [38], [39], [40], [41]]. The use of WNT inhibitors can sensitize cancer cells to chemotherapeutic agents [[42], [43], [44], [45]]. These studies suggest that manipulating the WNT pathway will be a promising modality for improving the efficacy of chemotherapy.

In addition to the use of conventional chemotherapy drugs, there has been a great deal of research into the use of WNT inhibitors to treat cancer, including the famous WNT inhibitor LGK974 [[46], [47], [48], [49]]. Considering the important role of WNT/β-catenin signaling in normal development, these available WNT inhibitors may lead to toxic effects in growth of bone, hair, gastrointestinal tract, and hematopoiesis, which limits their clinical application [[49], [50], [51], [52], [53]]. Therefore, developing novel WNT inhibitors is still urgently needed for enhancing the target and reducing the negative toxic effect. It may be feasible to overcome these limitations of the currently available WNT inhibitors from the following three aspects: Firstly, it is possible to screen for drugs with WNT inhibitory activity from FDA-approved drugs to develop novel WNT inhibitors. Secondly, new drugs with WNT inhibitory activity can be identified from natural products because of the abundant natural product resources. Finally, the existing compounds can be optimized by structure- and/or pharmacological-based modification for improving poor target, insolubility, low absorption, or toxicity, which will increase their potential for future applications.

### 3.1 New use of old drugs (FDA-approved)

Most of the FDA-approved antiparasitic agents, including artemisinin derivatives, quinine-related compounds, benzimidazole derivatives, Ivermectin, Niclosamide, and so on, have been applied clinically for a long time [[54], [55], [56]]. Although most of these FDA-approved drugs and natural products have multiple targets and may be not specific WNT pathway inhibitors, pharmaceutical scientists still keep exploring uncovered their novel antitumor mechanisms that can regulate the WNT signaling pathway since these drugs have been reported to influence the WNT signaling pathway. Increasing evidence has indicated that antiparasitic drugs have an amazing potential to be developed as WNT inhibitors. Studies have also reported that some antiparasitic species have the ability to inhibit WNT activity [[54], [55], [56], [57]]. Here, we reviewed the action mechanisms of these antiparasitic drugs in regulating the WNT signaling pathway (Table 1).

**Table 1:**
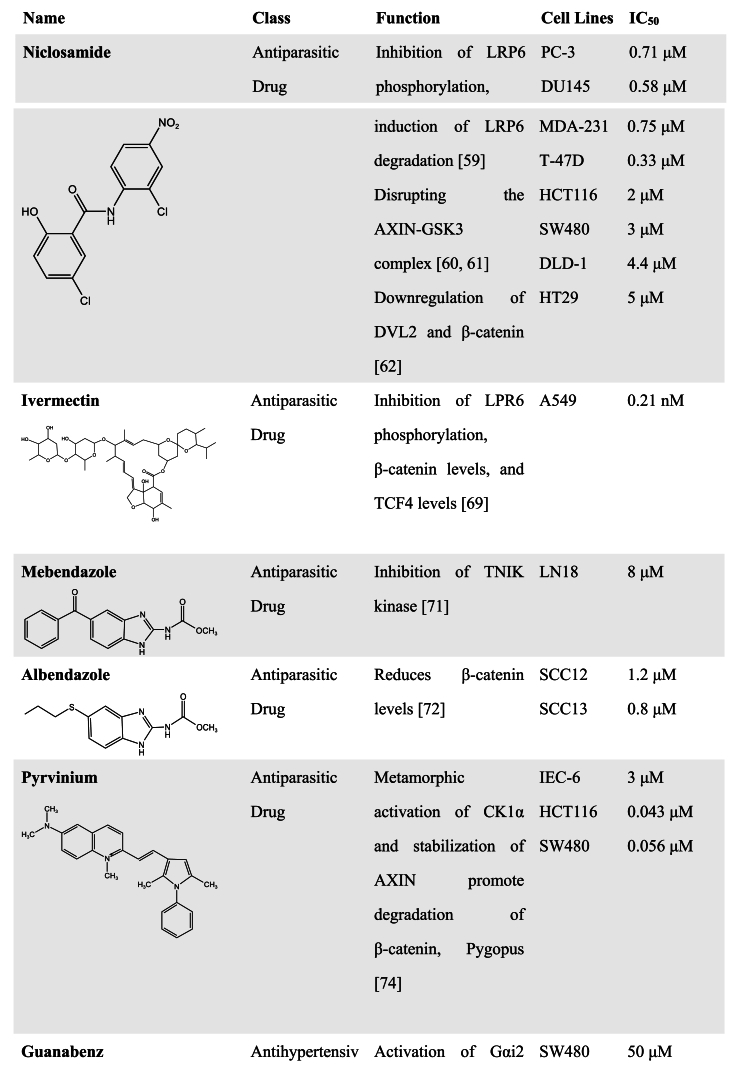

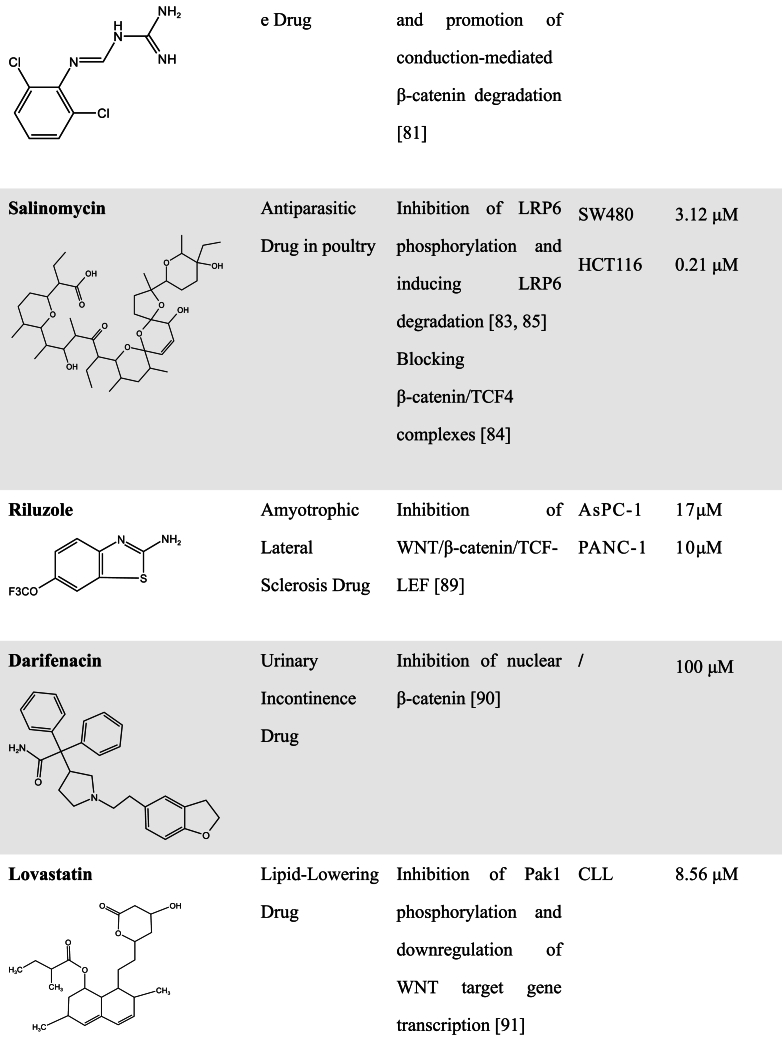

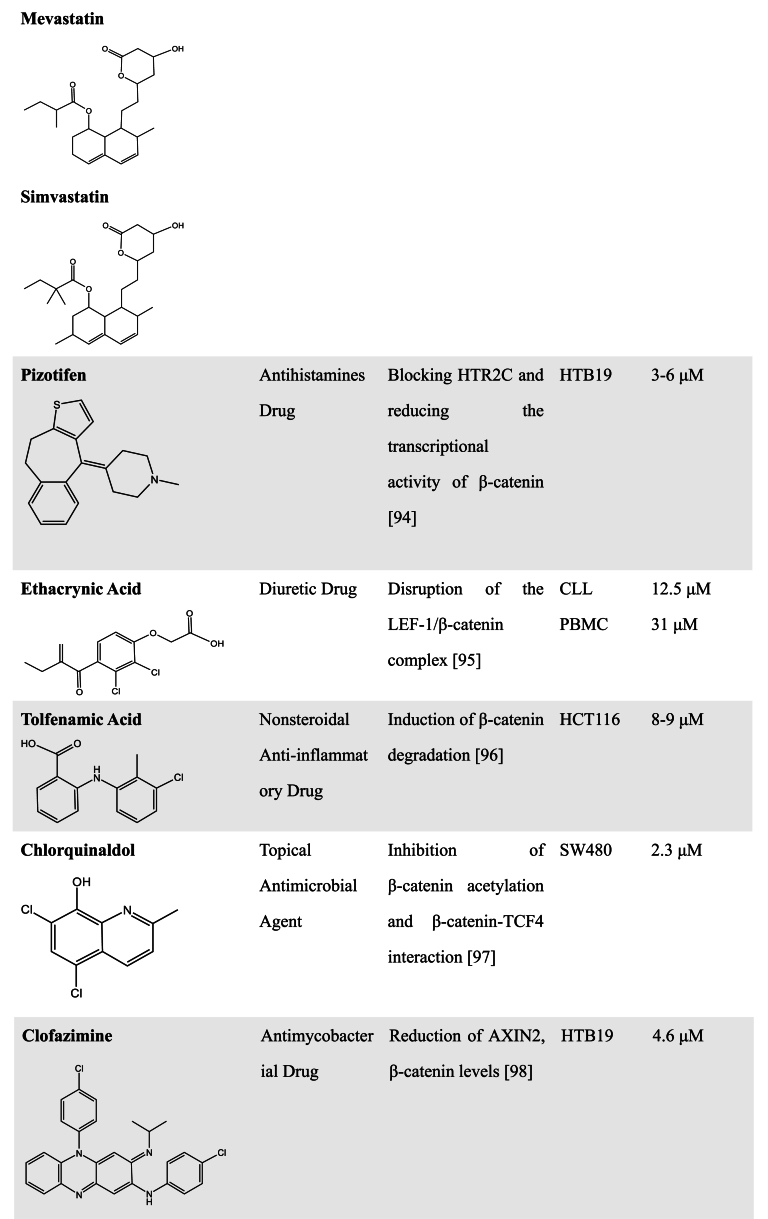
FDA-approved drugs with WNT inhibitory activity.

Niclosamide is an anthelmintic approved by FDA in 1982 for the treatment of parasitic infections [58]. It has been shown to inhibit the WNT/β-catenin signaling pathway through inducing LRP6 degradation [59]. Niclosamide could also bind directly to GSK3 and disrupt the AXIN-GSK3 interaction, resulting in inactivation of the WNT pathway [60,61]. In CRC with APC mutations, Niclosamide inhibits cancer cell growth by inhibiting the WNT/β-catenin pathway, which is attributed to Niclosamide-induced downregulation of DVL2 and β-catenin [62]. This is consistent with another study that WNT signaling is blocked by Niclosamide-induced autophagy in CRC [63]. In breast cancer, Niclosamide in combination with doxorubicin generates a synergistic antitumor effect and enhances the sensitivity of breast cancer cells to doxorubicin [64]. It is also observed that Niclosamide exhibits a killing effect on ovarian cancer via the inhibition of the WNT/β-catenin pathway [65].

The FDA-approved antiparasitic drug Ivermectin [[66], [67], [68]] has been recently reported to suppress tumor metastasis through inhibition of the WNT/β-catenin pathway. The increased β-catenin phosphorylation and the decreased LRP6 phosphorylation were observed after Ivermectin treatment in CRC cells. This study also indicated that Ivermectin inhibited the expression of metastasis-associated proteins by inhibiting WNT/β-catenin/integrin β1/FAK signaling [69].

Mebendazole and Albendazole, originating from the benzimidazole class of anthelmintics, are also thought to have inhibitory effects of WNT signaling. Mebendazole was identified as a TRAF2 and NCK-interacting kinase (TNIK) inhibitor after a comparative modeling screen. TNIK, characterized as an essential activator of WNT/β-catenin signaling, has been reported to bind to the promoters of the WNT target genes and induce directly TCF4 phosphorylation [70]. Therefore, Mebendazole functioned downstream of the WNT pathway to inhibit WNT signaling [71]. The study from Zhang et al. group indicated that Albendazole reduced protein levels of β-catenin and inhibited the stemness in human squamous cell carcinoma [72].

Pyrvinium [73], a quinoline-derived cyanine dye previously used in the treatment of pinworm infection, is an FDA-approved anthelminthic drug. Curtis et al. found Pyrvinium could bind all CK1 family members and selectively enhance the kinase activity of CK1α that inhibits activation of WNT/β-catenin signaling through inducing β-catenin phosphorylation and degradation. Moreover, Pyrvinium promoted the degradation of Pygopus, a WNT transcriptional component, leading to the decrease of cytoplasmic β-catenin and the increase of axial proteins [74]. It has been also found that Pyrvinium enhances the sensitivity of glioblastoma to temozolomide by suppressing the WNT/β-catenin pathway [75]. In almost half of all ER^+^ breast cancers, the oncogene phosphoinositide phosphatase INPP4B is highly expressed and can promote WNT/β-catenin activation. So, the ER^+^ breast cancer cells with INPP4B overexpression were more sensitive to Pyrvinium [76], which is ascribed to the inhibitory effect of Pyrvinium on the WNT/β-catenin pathway.

The antiparasitic drug Guanabenz is an orally active central α2-adrenoceptor agonist and is used for the treatment of hypertension [[77], [78], [79]]. Conductin is a key factor in the negative regulation of β-catenin and its polymerization shows high activity for suppressing WNT signaling [80]. Recently, Miete et al. found that Guanabenz has WNT pathway inhibitory effects and exerts tumoricidal potency by activating Gαi2-induced conductin condensation and promoting conductin-mediated β-catenin degradation in CRC cells [81].

Salinomycin, as an FDA-approved anticoccidial agent for poultry, was found to be efficient in killing mouse breast CSCs [82]. Lu et al. found that Salinomycin inhibited LRP6 phosphorylation and caused degradation of LRP6 protein, leading to inhibition of the WNT pathway [83]. Wang et al. further found that Salinomycin also has the function of blocking β-catenin/TCF4E complex formation [84]. A recent study also indicated the use of low concentrations of Salinomycin for osteoarthritis treatment by inhibiting the WNT/β-catenin signaling pathway, which is attributed to its inhibition on LRP6 phosphorylation [85].

In addition to antiparasitic drugs, many FDA-approved drugs have the potential to be developed as novel WNT inhibitors, and some of them will need to be further explored for their mechanism of action (Table 1). For example, Riluzole, an FDA-approved drug for treating amyotrophic lateral sclerosis, was found to inhibit the proliferation, migration, and invasion of cancer cells [[86], [87], [88]]. Its killing of melanoma was attributed to a reduction in expression of metabotropic glutamate receptor-1 (GRM1) that enhances β-catenin signaling [89]. A recent study showed that Riluzole induced apoptosis in pancreatic cancer cells and pancreatic CSCs without disturbing the viability of normal pancreatic epithelial cells. Mechanistic analyses indicated that Riluzole inhibited the hyperactive WNT/β-catenin/TCF pathway in pancreatic cancer cells, further downregulated the expression of WNT target genes, thus resulting in abnormal mitochondrial function and cancer cell apoptosis [90].

Darifenacin, a novel muscarinic receptor 3 (M3R) antagonist in clinical use, has been shown to cause downregulation of WNT ligand expression and nuclear accumulation of β-catenin in Osimertinib-regressed cells by inhibiting acetylcholine/M3R signaling and WNT signaling [91].

Shailes et al. generated APC-mutant clones using CRISPR-Cas9 editing and performed a compound screen to identify drugs that are synthetically lethal with mutant APC. It has been found that in comparison to the wild-type APC cells, the APC-mutant cells were extremely sensitive to Lovastatin, Mevastatin, and Simvastatin, belonging to the statin lipid-lowering family drugs. Statin treatment induced a decrease in WNT signaling and downregulation of the WNT target gene Survivin expression due to an increase in Rac1 activity in APC mutant cells [92]. Another FDA-approved anti-hypolipidemic agent Fenofibrate was also found to modulate both PPAR and WNT signaling [93], suggesting that it may be a potential WNT inhibitor in anticancer studies [94].

Using a zebrafish embryo screen model with gastrulation, Nakayama et al. found that the antihistamine Pizotifen could be used as an antagonist for serotonin receptor 2C (HTR2C) that activates WNT signaling and promotes EMT-mediated metastatic dissemination of human cancer cells. Pharmacological inhibition of HTR2C by Pizotifen could suppress the transcriptional activity of β-catenin by decreasing β-catenin accumulated in the nucleus [95]. Using a cell-based WNT reporter assay, the diuretic Ethacrynic Acid was identified as a WNT inhibitor by directly interacting with LEF-1 and disrupting the LEF-1/β-catenin complex, thereby leading to the inhibition of chronic lymphocytic leukemia growth [96].

The steroidal anti-inflammatory drug Tolfenamic Acid was found to inhibit colon cancer cell growth by downregulating Smad2 and promoting ubiquitin-proteasome-mediated β-catenin degradation, thereby inhibiting the WNT pathway. But the mRNA level and promoter activity of β-catenin were not affected by Tolfenamic acid, and overexpression of Smad2 reverted Tolfenamic acid-induced degradation of β-catenin [97]. Our previous study found that the antimicrobial drug Chlorquinaldol inhibited the interaction of β-catenin with TCF4 by decreasing β-catenin acetylation, thereby suppressing WNT target gene transcription and the stemness of cancer cells [98]. Another antimicrobial drug clofazimine has the activity to inhibit WNT signaling in breast cancer cells [99,100], which is possibly associated with the nuclear events triggered by the WNT pathway since clofazimine could not affect the nuclear translocation of β-catenin [100]. Further study showed that clofazimine exerted a good tumor-killing effect in other cancer cells such as CRC, HCC, and ovarian cancer, because of the high levels of WNT pathway activation in these cancer cells [101].

### 3.2 Natural products

A great number of scientific studies suggest that natural products as smart compounds can be effective to treat different cancer types. Molecules found in natural products have the ability to interact with more than one biological target. Therefore, searching for novel WNT inhibitors from natural products is a good approach [102]. In this section, we summarized natural compounds that exhibit inhibitory effects on WNT pathway activity (Table 2), further emphasizing the immense potential of exploring novel WNT signaling inhibitors from natural products.

**Table 2:**
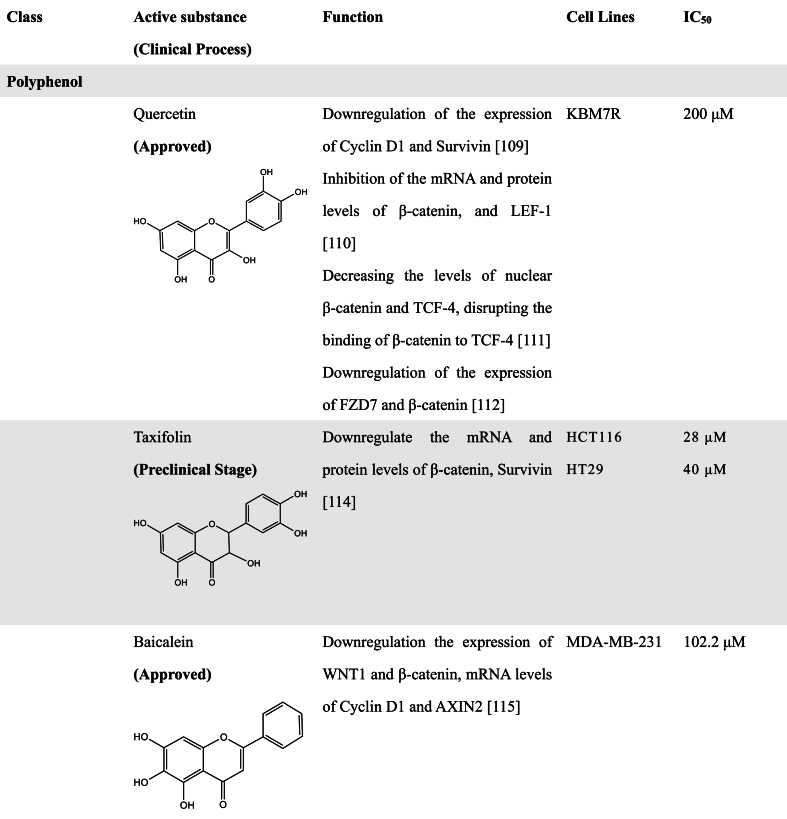

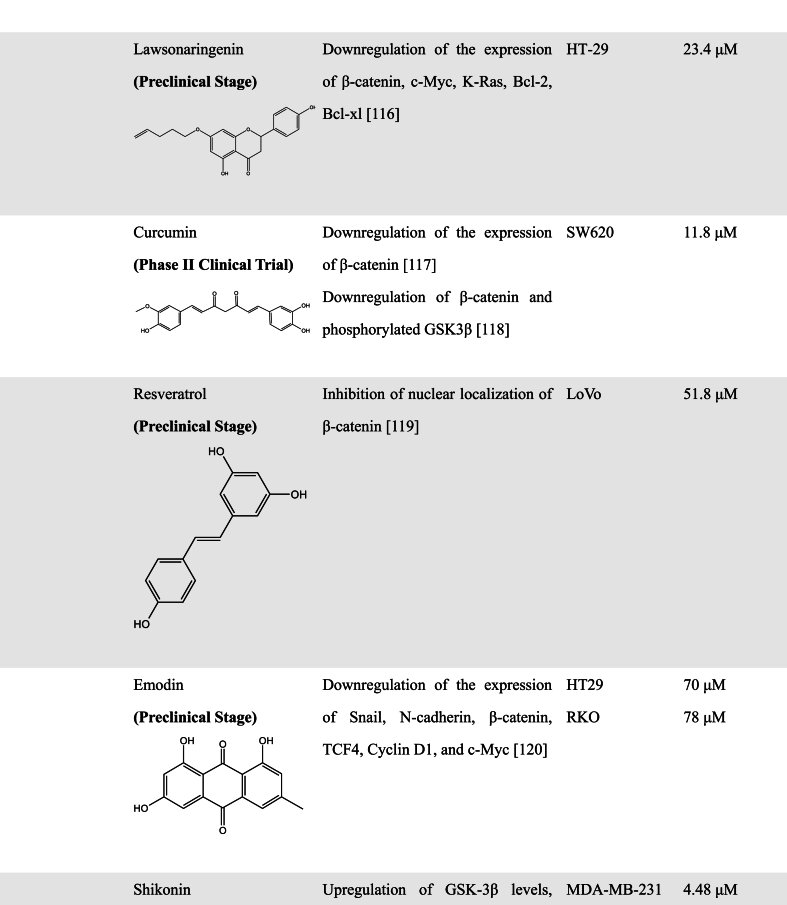

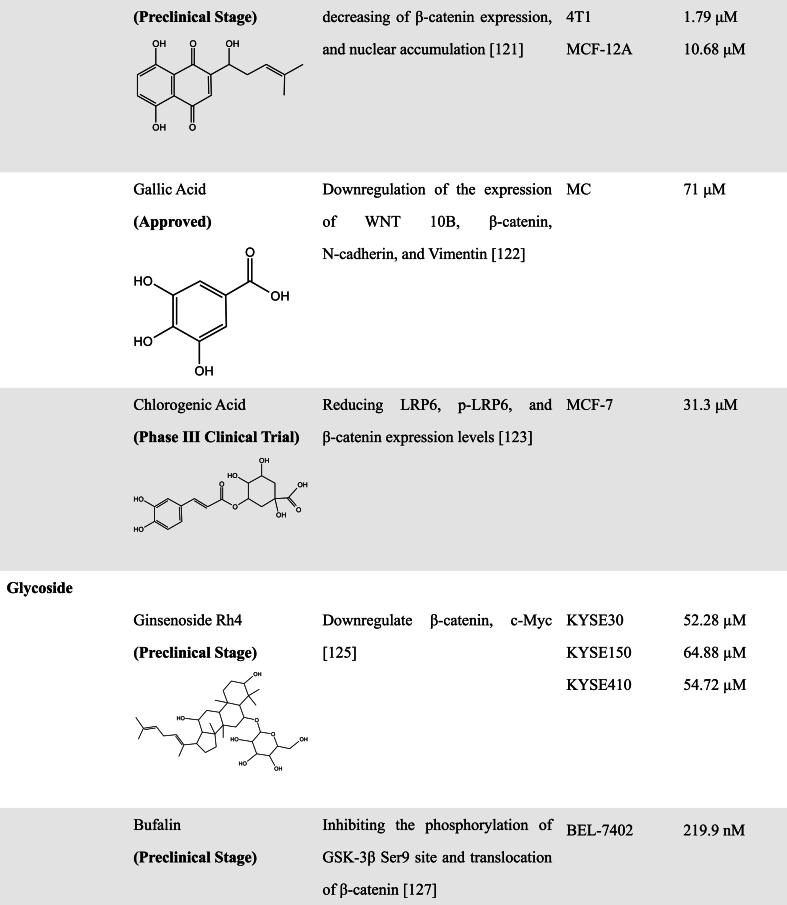

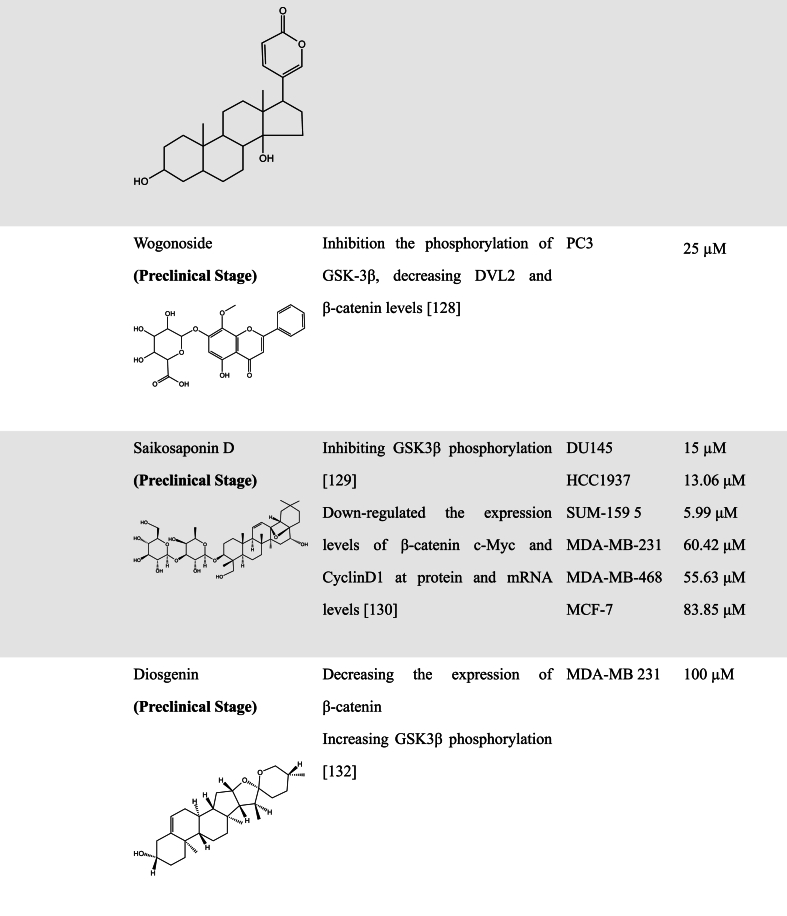

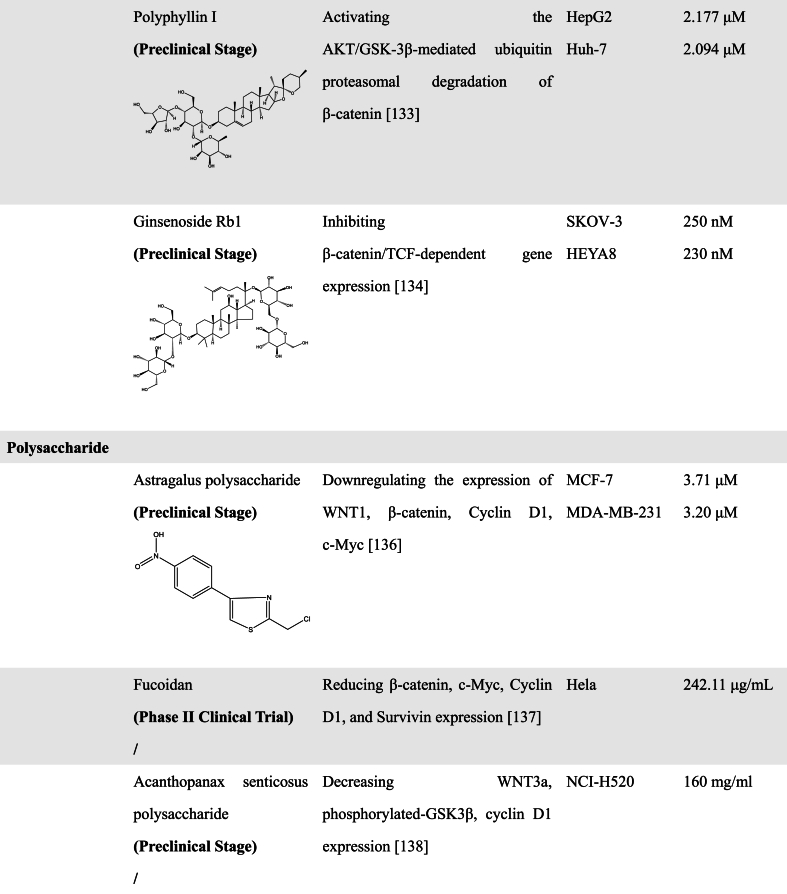

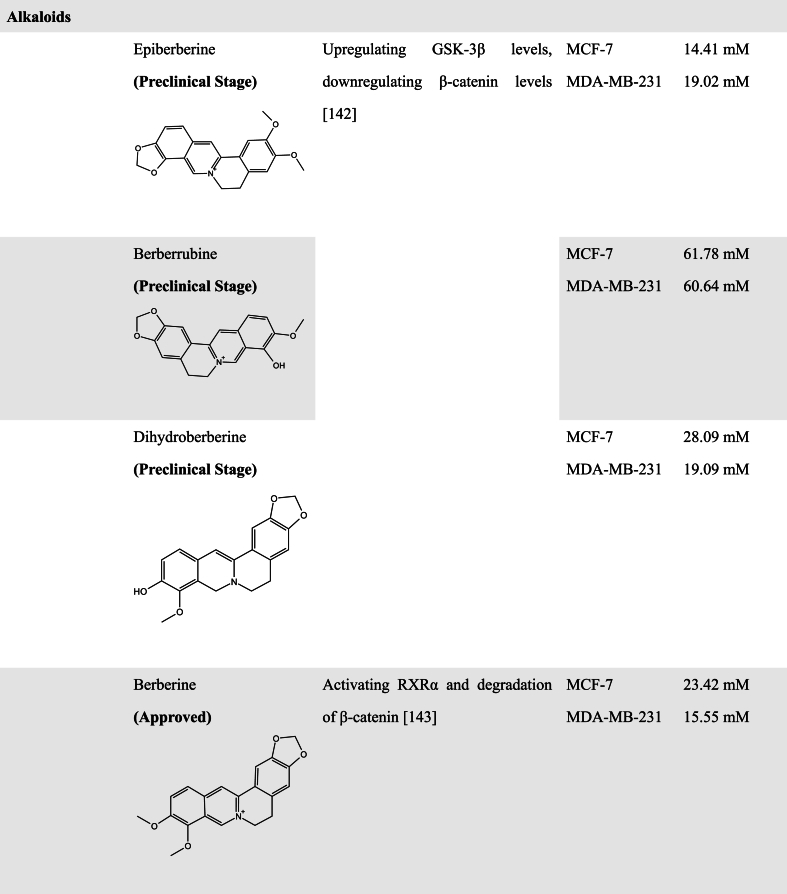

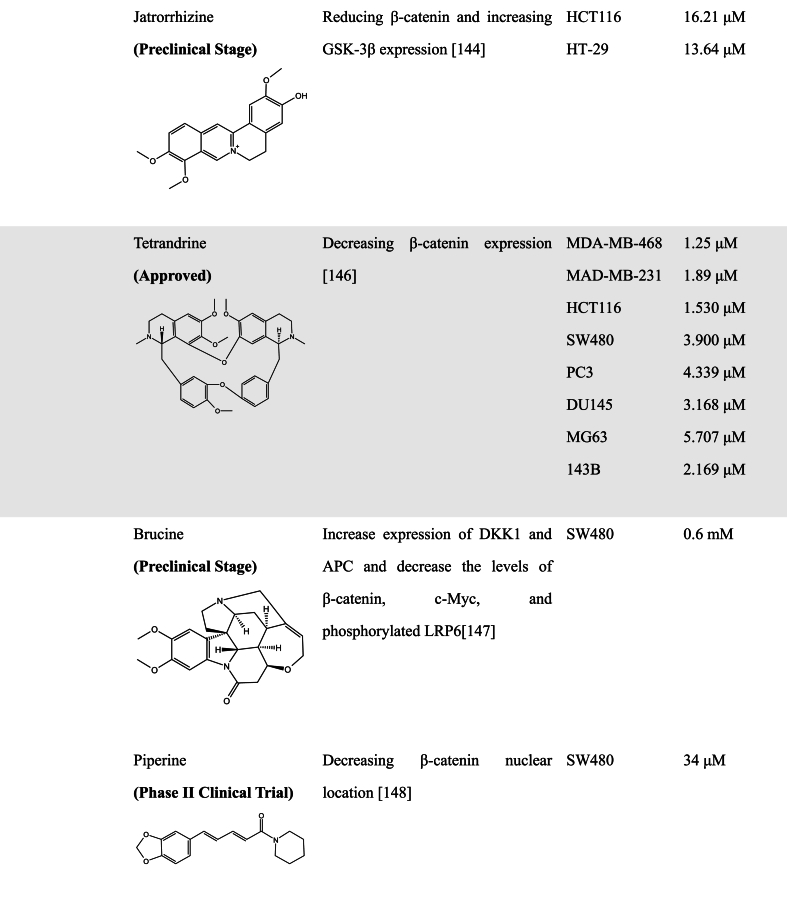

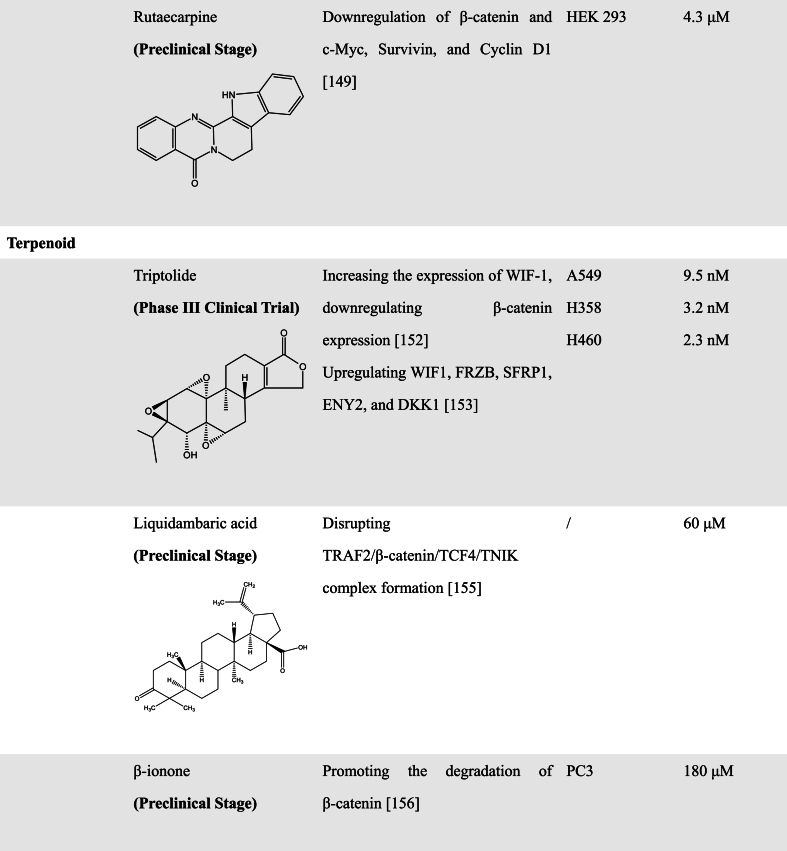

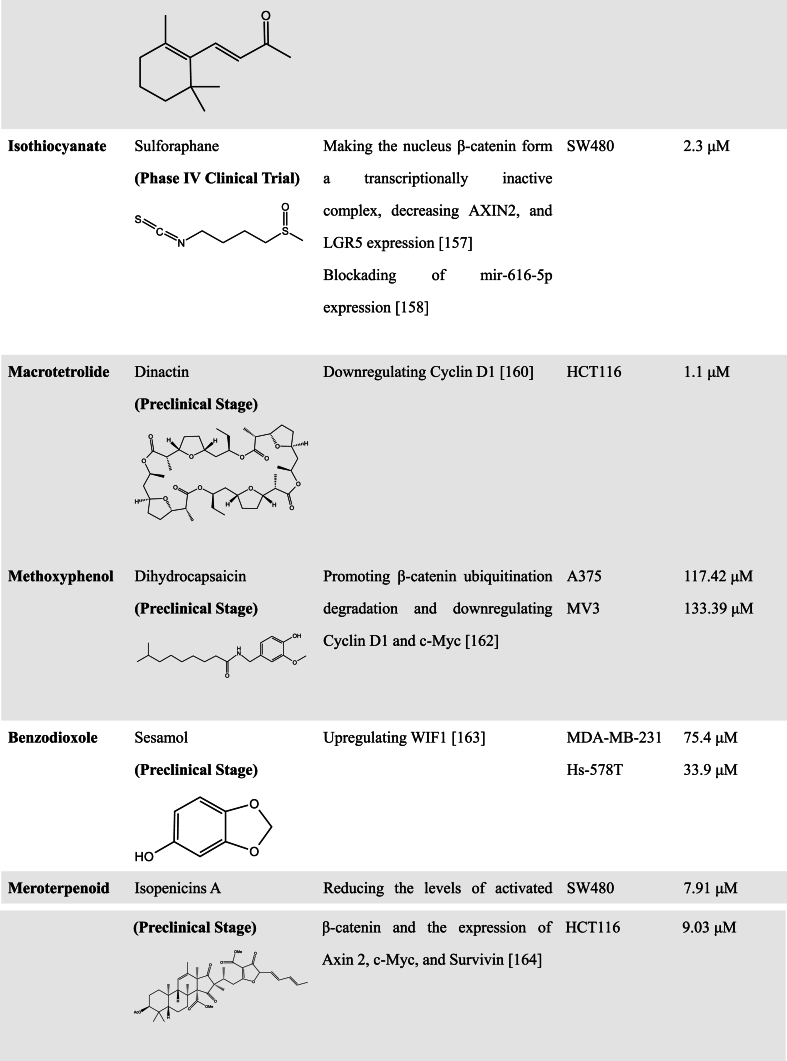
Natural Products with WNT inhibitory activity.

#### 3.2.1 Polyphenolic compounds

Natural polyphenols are major secondary metabolites of plants ranging from small molecules to polymers, and are potential candidates for the discovery of anticancer drugs [103,104]. Based on chemical structures, natural polyphenols are divided into different categories, such as flavonoids (60 %), phenolic acids (30 %), lignans, and stilbene [105]. Among them, flavonoids, a large group of polyphenolic compounds, are ubiquitous in plants [106,107]. Flavonoids possess a variety of biological activities, including antioxidant, antitumor, and antiviral effects [108]. Several studies have pointed out that flavonoids also have a regulatory effect on the WNT pathway [109,110]. For example, Quercetin, a flavonoid compound, has the effect of inhibiting the WNT pathway in CRC cells and chronic granulocytic leukemia [111,112]. Chi et al. found that Quercetin inhibited the WNT pathway in CRC cells by decreasing the levels of nuclear β-catenin and TCF-4, as well as disrupting the interaction between β-catenin and TCF-4 [113]. Chen et al. found that Quercetin exerted the ability to reverse multidrug resistance by targeting FZD7/β-catenin to inhibit WNT signaling [114]. Another study has found that Quercetin can modulate the expression of APC and β-catenin and attenuate the development of colorectal tumors induced by 1,2 dimethylhydrazine [115]. An analog of Quercetin, dihydroquercetin (Taxifolin, TAX) was also found to decrease the mRNA and protein levels of β-catenin, and downregulate the expression of AKT and Survivin in vitro and in vivo [116].

Ma et al. indicated that Baicalein, extracted from *Scutellaria baicalensis*, could inhibit breast cancer proliferation, migration, and invasion in a time- and dose-dependent manner. Further experiments showed that Baicalein could downregulate the expression of WNT1 and β-catenin, leading to the decrease of WNT/β-catenin downstream target genes such as vimentin and snail [117]. Areeba's team discovered for the first time that Lawsonaringenin (LSG), a bioactive flavonoid extracted from *henna* leaves, exerted anti-CRC activity by arresting the cell cycle in the S phase. Except for downregulation of oncogene K-Ras and anti-apoptotic proteins Bcl-2 and Bcl-xL, LSG could also elevate the level of β-catenin phosphorylation and decrease oppositely the expression of non-phosphorylated β-catenin and its downstream signaling target c-Myc [118], suggesting its inhibitory effect on WNT/β-catenin signaling pathway.

In addition to some flavonoid compounds, some other natural polyphenols also have WNT pathway inhibitory activity. Previous studies have demonstrated that the plant polyphenol Curcumin, extracted from turmeric of the ginger family, can reduce significantly the level of β-catenin expression and its anti-tumor activity is closely associated with the inhibition of the WNT/β-catenin signaling pathway. Zhang et al. found that Curcumin could promote the expression of naked cuticle homolog 2 (NKD2), an inhibitor of the WNT signaling pathway, and knockdown of NKD2 could increase the expression of β-catenin, TCF4, and vimentin protein expression and decrease E-cadherin expression [119]. This is also verified by another study that Curcumin inhibits TPA-induced migration of HCC cells in vitro as well as tumor growth in vivo by inhibiting the WNT pathway, which is related to the reduction of β-catenin and phosphorylated GSK3β [120].

The antioxidant Resveratrol is isolated from *C. tigrinum* and has been reported to function as an antitumor drug by some mechanisms such as reducing blood viscosity, preventing the development of cancer, and so on. Increasing evidence has indicated that Resveratrol could inhibit the WNT/β-catenin signaling by modulating long non-coding metastasis-associated lung adenocarcinoma transcript 1 (RNA-MALAT1). MALAT1, often overexpressed in CRC, could increase nuclear localization of β-catenin, and the increased effect was reversed by Resveratrol in LoVo cells [121]. Emodin, widely found in Rheum, Polygonum, and Cassia, has been also shown to inhibit the WNT/β-catenin signaling pathway by downregulating the expression of related WNT target genes TCF4, cyclin D1, and c-Myc, thereby resulting in the inhibition of epithelial-mesenchymal transition (EMT) as well as invasion and migration of colon cancer cells in vitro and in vivo [122].

Shikonin, a primary constituent of *Lithospermum erythrorhizon,* is a derivative of naphthoquinone, which has been shown to upregulate the GSK-3β levels, leading to enhanced phosphorylation levels of β-catenin and the decrease of β-catenin nuclear accumulation. Meanwhile, E-cadherin upregulation and vimentin downregulation were observed after Shikonin treatment, suggesting Shikonin is a potential candidate for a novel anticancer drug against TNBC metastasis by targeting EMT [123].

Gallic Acid is a natural polyphenolic compound derived from *Rhus chinensis Mill.* It has strong antitumor effects and can affect multiple cellular pathways associated with cancer development and progression. Recently, Liao et al. demonstrated that Gallic Acid could inhibit the WNT/β-catenin signaling pathway and EMT process to attenuate gastric precancerous lesions [124]. Another polyphenol compound Chlorogenic Acid directly interacted with the co-receptor LRP6 and reduced the expression levels of LRP6, phosphorylated LRP6, β-catenin, thus inhibiting the WNT/β-catenin signaling pathway in MCF-7 cells [125].

#### 3.2.2 Glycoside compounds

Glycosides are derivatives of sugars, most of which are found in nature, primarily in plants, and have a variety of biological activities. Ophiopogonin B was found to have the ability to enhance the interaction between AXIN and β-catenin and reduce β-catenin protein translocation to inhibit the WNT/β-catenin signaling pathway, which reduces the migration and invasion of non-small cell lung cancer (NSCLC) cells [126]. Recently, Chen et al. discovered that Ginsenoside Rh4, a ginseng-derived substance, has the ability to inhibit the metastasis of esophageal squamous cell carcinoma. Further experiments demonstrated that Ginsenoside Rh4 could inhibit the WNT/β-catenin pathways through downregulating the expression of β-catenin and c-Myc [127].

Bufalin is one of the main components extracted from toad venom and has been confirmed to have anticancer activity. Moreover, it can reverse the multi-drug resistance of HCC cells and enhance HCC cells sensitization to sorafenib, leading to the suppression of cell proliferation [128]. Gai et al. found that Bufalin could inhibit GSK-3β phosphorylation at Ser9 site in HCC cells BEL-7402, and decrease the expression of β-catenin and WNT target gene Cyclin D1 to control the EMT, invasion, and metastasis of HCC cells [129].

Wogonoside, an effective component of *Scutellaria baicalensis*, was found to inhibit the phosphorylation of GSK-3β at Ser9 site and decrease DVL2 and β-catenin levels [130]. Similarly, Saikosaponin D (SSD), one of the prominent triterpenoid saponins, was also found to block the WNT/β-catenin signaling pathway by inhibiting GSK3β phosphorylation [131]. Both Wogonoside and SSD exerted the function of inhibiting the EMT process as well as migration and invasion in prostate cancer cells. Notably, SSD could suppress CSCs phenotypes such as self-renewal ability. Another study also discovered that SSD significantly inhibited β-catenin and its downstream target genes in TNBC cells. Based on the docking of SSD to the crystal structure of β-catenin, the authors further revealed that SSD could interact directly with β-catenin by hydrogen bonds and hydrophobic interaction [132]. A recent study by Wang et al. also found that Wogonoside also has inhibitory activity against cutaneous squamous cell carcinoma stemness through inactivating both PI3K/AKT and WNT/β-catenin pathways [133].

Diosgenin (DG), Polyphyllin (PPI), and Ginsenoside Rb1 (Rb1) have also been found to have the ability to inhibit CSCs by inhibition of WNT/β-catenin pathways. After treatment with DG, the viability of breast CSCs was inhibited and the stemness markers CD44 and ALDH were downregulated [134]. PPI, a steroidal saponin isolated from *Paris polyphylla*, was found to activate the AKT/GSK-3β-mediated ubiquitin proteasomal degradation of β-catenin and attenuated the prooncogenic effect of liver CSCs [135]. Rb1 and compound K (an end product of intestinal bacterial metabolism of Rb1) can downregulate β-catenin/TCF-dependent transcription and expression of target genes ATP-binding cassette G2 and P-glycoprotein [136].

#### 3.2.3 Polysaccharide compounds

Polysaccharides are natural polymers that are widely found in animals, plants, and microorganisms and have a variety of biological activities such as antitumor, antiviral, antioxidant, and immunomodulatory [137]. Polysaccharide compounds also have the potential to become WNT inhibitors. Studies have reported that some polysaccharide compounds exhibit inhibitory effects on the WNT/β-catenin signaling pathway. Here, we review the mechanisms of some polysaccharide compounds associated with the WNT/β-catenin signaling pathway.

Astragalus polysaccharide (APS) is one of the main bioactive components extracted from the root of Astragalus Membranaceus. Yang et al. demonstrated that APS could inhibit breast cancer migration and invasion by a specific mechanism that APS decreased the expression levels of snail and vimentin, and increased E-cadherin expression, which was closely related to APS-induced inhibition of WNT/β-catenin signaling, since APS downregulated WNT1, β-catenin and downstream target expression [138].

Xue et al. have studied the relationship between Fucoidan, a sulfated polysaccharide derived from brown algae, and β-catenin in mouse breast cancer. Experimental results showed that Fucoidan reduced the β-catenin levels and downregulated the expression of downstream target genes such as c-Myc, Cyclin D1, and Survivin, suggesting that the anti-breast cancer effects of Fucoidan are closely associated with the downregulation of WNT/β-catenin signaling [139].

Sun et al. found that Acanthopanax senticosus polysaccharide (ASPS) derived from the roots of Acanthopanax could exert the ability to inhibit the proliferation, invasion, and migration of human NSCLC cells. Mechanistic analyses showed that ASPS downregulated the levels of Cyclin D1 and inhibited the EMT process by promoting GSK3β-mediated β-catenin degradation to suppress the WNT/β-catenin pathway. It was also observed that ASPS decreased the levels of phosphorylated GSK3β, not the total GSK3β in human NSCLC cell line, indicating that the inhibitory effect of ASPS on proliferation and metastasis may be dependent dominantly on its regulation of GSK3β activity [140].

A study investigating the effect of Apple Polysaccharide (AP) on modulating intestinal microbiota disease to prevent colitis-associated CRC (CACC) revealed that AP inhibited the activation of the classical WNT pathway by suppressing β-catenin translocation from the cytoplasm to the nucleus in CACC mice. Interestingly, treating HT-29 and HCT116 colorectal cells with AP did not inhibit the growth of CRC cells and had no significant effect on the expression of regulatory proteins associated with the WNT/β-catenin signaling pathway [141]. These studies suggest that the screening of WNT inhibitors should not only be based on the cellular level but should also be considered at the level of living organisms or ecosystems.

#### 3.2.4 Alkaloids

Alkaloids are a class of nitrogenous basic organic compounds found in nature and have a variety of biological activities [142]. Several studies have also reported that some alkaloids have WNT pathway inhibitory activity. Isoquinoline alkaloids are one of the most abundant alkaloids in the plant [143]. Berberine alkaloids belong to the class of isoquinoline alkaloids used in traditional Chinese medicine that are mainly isolated from several members of the Berberidaceae [144]. It has been reported that different berberine alkaloids have different effects on anti-cancer treatment. For example, Berberrubine and Dihydroberberine could inhibit the proliferation of breast cancer cells. However, the cell cycle of MCF-7 cells was arrested at the G2/M phase after berberine alkaloids treatment, and in MDA-MB-231 cells berberine alkaloids induced the cell cycle arrest in G0/G1 and G2/M phases. It was also observed that Epiberberine, Berberrubine, and Dihydroberberine could upregulate GSK-3β levels, downregulate β-catenin levels, and reverse EMT properties, but berberine exhibited no effect on the invasion of MDA-MB-231 cells [144]. Another study by Ruan et al. in KM12C colon cancer cells showed that berberine promoted the interaction of receptor retinoid X receptor alpha (RXRα) with nuclear β-catenin, leading to c-Cbl-mediated degradation of β-catenin, thereby inhibiting the proliferation of colon cancer cells, which was attributed to the direct binding of berberine with a unique region comprising residues Gln275, Arg316 and Arg371 in nuclear RXRα [145], indicating berberine as an RXRα activator.

Jatrorrhizine (JAT), a natural protoberberine alkaloid, has been demonstrated to possess detoxification, bactericidal, hypoglycemic, and hypolipidemic effects. JAT has a similar parent structure to Berberine. Wang et al. demonstrated that JAT could inhibit the proliferation of HCT116 and HT29 cells, inducing cell cycle arrest in the S phase. Meanwhile, it has been found that JAT could inhibit the WNT signaling pathway by reducing β-catenin and increasing GSK-3β expression [146]. TNIK is a tumor target protein which high expression is closely related to poor prognosis. Previous studies pointed out that JAT had good affinity and interaction with TNIK by molecular docking, and experimental results indicated JAT as a potential inhibitor of proliferation and metastasis of breast cancer cells through the inhibition of TNIK-regulated WNT/β-catenin signaling and EMT expression [147]. However, its activity for targeting TNIK needs to be further validated.

Tetrandrine (TET) is a bisbenzylisoquinoline alkaloid extracted from the root of *Stephania tetrandra* (or *hang fang ji*) and has been used as an effective antihypertensive and antiarrhythmic agent in Chinese medicine. It has been reported that TET exhibits anticancer activity via targeting certain regulatory signals that are involved in proliferation and apoptosis. He et al. found that TET exhibited anticancer activity comparable with camptothecin, vincristine, paclitaxel, and doxorubicin, and generated synergistic anticancer activity with 5-FU in colon cancer. Moreover, treatment with TET induced a decrease in β-catenin expression, suggesting that TET possesses WNT inhibitory ability. It is noteworthy that β-catenin knockout decreased HCT116 cells sensitive to TET-induced inhibition of cell proliferation, cell viability, and xenograft tumor growth [148], indicating that the anticancer activity of TET may be dependent on its inhibition on β-catenin expression.

Brucine and Strychnine are the main active ingredients of nux vomica, which are widely used in the clinical treatment of various tumors. Ren's study reported that Brucine and Strychnine could increase the expression of DKK1 and APC and decrease the levels of β-catenin, c-Myc, and phosphorylated LRP6 in CRC cells and tumor tissues, which reveals that Brucine and Strychnine induce cell apoptosis and exhibit antitumor activity of CRC probably through suppressing WNT/β-catenin signaling [149].

Piperine is a true alkaloid, derived from lysine, responsible for the spicy taste of black pepper (*Piper nigrum*) and long pepper (*Piper longum*). Studies have shown that Piperine has a wide range of pharmacological properties such as anticonvulsive activity, antioxidant activity, anti-inflammatory and anticancer activity. Effects of Piperine on CRC were reported to inhibit cell proliferation and induce an apoptotic program by endoplasmic reticulum stress. De Almeida's study further demonstrated that Piperine could suppress the WNT signaling pathway by decreasing the β-catenin nuclear location in HCT116 cells, leading to the inhibition of cell proliferation and migration. Importantly, Piperine exhibits no effect on the proliferation and migration of non-tumorigenic intestinal cell line IEC-6 [150].

Rutaecarpine, an alkaloid obtained from *Evodia rutaecarpa*, has been previously reported as an anti-inflammatory agent. It has been shown that Rutaecarpine could induce G_0_/G_1_ cell cycle arrest and apoptotic cell death in CRC cell Ls174T, which is closely associated with its inhibition effect on the WNT/β-catenin signaling pathway, since Rutaecarpine treatment resulted in downregulation of β-catenin and WNT target genes, further inhibiting cell migration, invasion, and EMT processes [151].

#### 3.2.5 Terpenoids

Terpenoids are the largest class of natural products and a rich candidate compound pool for drug discovery [152]. Recent efforts into the research and development of anti-cancer drugs have led to the identification of a variety of terpenoids that inhibit cancer cell proliferation and metastasis via various mechanisms. Here, we intend to summarize recent progress of some naturally derived terpenoids that function as the inhibitor of the WNT signaling pathway in the investigation of anti-cancer activities.

Triptolide (TP) is a diterpenoid triepoxide and the major active component derived from *Tripterygium wilfordii* [153]. It has been reported to possess potent anticancer, anti-inflammatory, and immunosuppressive properties. A study by Mao et al. showed that the anticancer activity of TP was regulated by the WNT, p53, and nuclear factor-κB (NF-κB) signaling pathways. TP treatment could upregulate cellular tumor antigen p53 expression, and downregulate matrix metalloproteinase-9 (MMP-9) and phosphorylated NF-κB levels. Moreover, TP could induce the demethylation of the WNT inhibitory factor-1 (WIF-1) promoter and thereby downregulate β-catenin expression to inhibit WNT signaling in NSCLC. Silencing WIF-1 reserved the upregulation of WIF-1 induced by TP and activated WNT signaling. Notably, compared with the control cells, knockdown of WIF-1 did not completely recover the levels of p53, MMP-9, and phosphorylated NF-κB in A549 cells treated with TP [154]. Previous study has also indicated that TP derivative MRx102 upregulated WIF1 in lung cancer cell lines through decreasing methylation at the WIF1 promoter and increasing the unmethylated population in both A549 and H460 cells, exhibiting the inhibitory effect on the WNT pathway. Similar results were obtained by another study that multiple WNT inhibitory factors including WIF1, FRZB, SFRP1, ENY2, and DKK1 were upregulated in lung cancer cells in A549 after TP treatment [155]. However, the authors claimed that TP-induced apoptosis was closely associated with global epigenetic changes to histone 3 (H3), and TP had no effect on DNA methylation status at any of the CpG islands located in the promoter regions of these WNT inhibitory factors [155]. Therefore, the effect of TP on the status of WIF-1 methylation is still unclear and needs to be further investigated.

Tumor necrosis factor receptor-associated factor 2 (TRAF2) is an adaptor molecule that has been associated with multiple receptor-specific functions in tumor initiation and progression [156]. Yan et al. identified TRAF2 as a positive regulator of the canonical WNT signaling pathway [157]. TRAF2 could interact with the first 32 residues of β-catenin at the N-terminal to promote the formation of β-catenin/TCF4 complex. Using a TGC (TCF driving GFP and SV40 driving mCherry) reporter in EPT1 cells, it has been found that Liquidambaric acid (LDA), derived from betulinol, pentacyclic styrene, was identified from a library of 2000 natural products as a candidate inhibitor of oncogenic WNT/β-catenin signaling. Mechanically, LDA directly targets TRAF2 and disrupts TRAF2/β-catenin/TCF4/TNIK complex formation, thereby inhibiting WNT/β-catenin signaling in CRC [157].

β-ionone is a terminal cyclic analog of carotenoids that exerts antitumor effects in various cancer. Recently, it has been reported that β-catenin was downregulated in prostate cancer cells after β-ionone treatment. Further mechanistic analyses showed that β-ionone could promote the ubiquitination and degradation of β-catenin to inhibit the WNT pathway, thereby suppressing the migration, invasion, and EMT process of prostate cancer cells [158].

#### 3.2.6 Other natural products

In addition to the above compounds, there are still a variety of natural products with WNT inhibitory functions. For example, Sulforaphane (SFN), a natural isothiocyanate, makes the nucleus β-catenin form a transcriptionally inactive complex, which inhibits WNT signaling and functions to induce cell death and inhibit proliferation in CRC cells [159]. Another study on SFN found that it has the ability to inhibit the migration and invasion of NSCLC. The mechanism is that SFN reduces miR-616-5p levels through histone modification, and inactivates the GSK3β/β-catenin signaling pathway, further leading to the inhibition of the WNT pathway [160].

Dinactin, a macrolide isolated from Streptomyces pusillus strain AS13 by Hussain et al., exhibited strong anti-microbial activity against bacterial pathogens including Mycobacterium tuberculosis. It has been also found to exert marked anti-tumor potential (IC50 ∼ 1.1–9.7 μM) and inhibit the proliferation of several cancers with less toxicity to normal cells (IC50∼80 μM) [161]. Further studies revealed that this compound inhibited the WNT signaling pathway and induced cell cycle arrest at the G_1_/S phase with downregulation of cyclin D1 in HCC and CRC cells. Moreover, its inhibition effect on TopFlash (specific reporter of WNT signaling) activity was more sensitive than that of Salinomycin in HCT-116 and HepG2 cells [162].

A previous study by Huang et al. showed that pearl shell meat hydrolysate has dual activities of antioxidation and tyrosinase inhibition. Recently, the authors found that the three active peptides FLF, SPSSS, and WLL isolated from pearl shell flesh hydrolysate extraction all have high tyrosinase inhibitory activities and exhibit the ability to inhibit the WNT/β-catenin, MAPK, and MC1R/α-MSH signaling pathway. It was also observed that the expression of WNT4 and β-catenin were downregulated after these three active peptides treatment [163].

Dihydrocapsaicin (DHC), one of the main active ingredients in chili peppers, has been reported to exert anti-cancer effects in various cancers. Shi et al. reported that DHC could inhibit the proliferation, migration, and invasion of melanoma cells. Exogenous β-catenin overexpression retrieved DHC-induced suppression of cell proliferation, migration, and invasion. With β-catenin downregulation, its downstream target genes such as Cyclin D1, c-Myc, were decreased in melanoma A375 and MV3 cell lines after DHC treatment. Mechanistically, DHC promoted β-catenin ubiquitination degradation by upregulating the expression of β-TrCP to inhibit the WNT pathway in melanoma [164].

Sesamol, a kind of phenolic ingredient enriched in sesame seeds and sesame oil, has been shown to inhibit TNBC proliferation and metastasis through WIF1-induced inactivation of WNT/β-catenin signaling since sesamol induces the promoter region demethylation of WIF1 and promotes its expression [165].

Tang et al. extracted three compounds, Isopenicins A-C, from Penicillium sp. sh18. Using the SuperTopflash luciferase system, they found that Isopenicins A exhibited WNT inhibitory activity. Further investigation revealed that Isopenicins A is capable of reducing the levels of activated β-catenin and expression of WNT target genes, such as Axin 2, c-Myc, and Survivin, thereby inhibiting the growth of CRC cells [166].

Although the mentioned above drugs all exhibit the inhibitory effects on the WNT signaling pathway, studies about these drugs still remain at the in vitro level, even some of them lack the verification of animal experiments, and the development of preclinical studies is also an urgent problem. Moreover, there are still many questions about the properties of these drugs, such as the poor target, toxic side effects, and lack of metabolic information, etc. Based on the existing knowledge of these drugs, more researchers familiar with pharmacochemistry and pharmacology need to work together to develop novel drugs with better solubility, lower toxicity and better absorption, explore their anti-tumor mechanisms in targeting WNT signaling pathway, which provides more optional therapeutic drugs for clinical research.

### 3.3 Structural optimization to find novel WNT inhibitors

Although most drugs including natural products have been extensively investigated as potential anticancer agents in both phenotypic and target-focused approaches, their direct therapeutic application may be impeded by insufficient efficacy, unacceptable pharmacokinetic properties, undesirable toxicity, or poor availability, and structural optimization will be necessary. So far, no drugs targeting WNT signaling have been available in clinic [167]. Therefore, using the existing drugs including natural products as lead templates, their structural optimization is subjected to generate clinically useful structures, which is helpful for improving drug efficacy and providing a scientific basis for their clinical practice [168]. Here, we review recent progress of the optimization of some drugs targeting the WNT signaling pathway, and briefly introduce the methods of these drugs optimization and the effects of these optimization on their antitumor activities, expecting to provide certain research direction for drug optimization in the future.

#### 3.3.1 Optimize physical and chemical properties

**Fig. 2:**
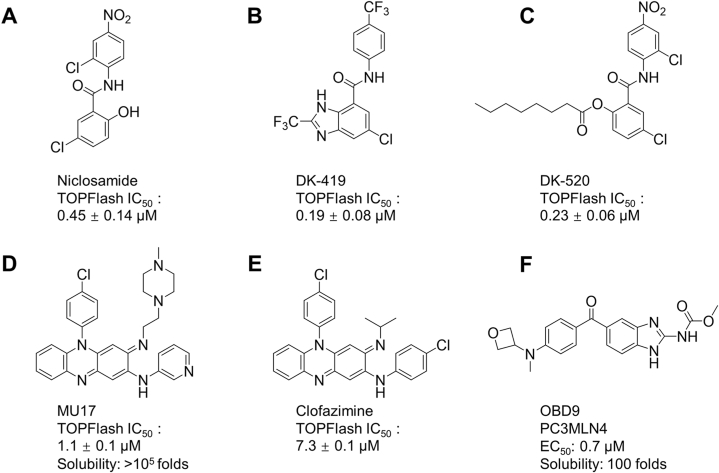
Optimization of physical and chemical properties for Clofazimine, Niclosamide, Benzimidazole.

To improve the disadvantages of poor absorption and rapid metabolism of Niclosamide (Fig. 2A), Wang et al. used trifluoromethyl substituted 4′-nitro to obtain the target compound DK-419 (Fig. 2B). Similar to Niclosamide, DK-419 with multifunctional activity and improved pharmacokinetic properties could induce Frizzled internalization and inhibit WNT/β-catenin signaling [169]. The Niclosamide acyl derivative DK-520 (Fig. 2C) was obtained by Robert et al. according to a similar principle [170]. These two derivatives have a similar function to Niclosamide and exhibit the inhibitory effect on WNT signaling with an IC_50_ of 0.19 ± 0.08 μM (DK-419), 0.23 ± 0.06 μM (DK-520) in the TOPFlash assay, comparable to Niclosamide (IC_50_ of 0.45 ± 0.14 μM).

Alexey et al. optimized the structure of clofazimine to obtain the target compound MU17 (Fig. 2D) with higher water solubility and potency as a WNT inhibitor. Compared to clofazimine (Fig. 2E), the water solubility of MU17 was increased at least by a factor of ∼10^5^. Clofazimine showed a WNT-Inhibitory activity with IC_50_ 7.3 ± 0.1 μM, and MU17 displayed a 7-fold better potency against WNT signaling (IC_50_ 1.1 ± 0.1 μM) and reduced the side effects of clofazimine skin staining. Similar to clofazimine, MU17 could not affect the WNT3a-induced decrease in phosphorylated-β-catenin nor the total β-catenin accumulation. The author concluded that their molecular target may be downstream of nuclear translocation of β-catenin [171]. Further study will be still necessary to identify the nuclear WNT pathway target of them.

Cheong et al. designed and synthesized an oxetane derivative of the benzimidazole compound, OBD9 (compound 18) (Fig. 2F), with a 100-fold increase in water solubility compared to the original compound benzimidazole [172]. Further experiments by Zhou et al. demonstrated that OBD9 could inhibit TNIK kinase activity, and promote TNIK degradation through an autophagic mechanism that is mediated by LAMP-mediated lysosomal degradation in CRC cells, thereby blocking TNIK-induced activation of WNT/β-catenin signaling. Moreover, OBD9 had strong anticancer activity as an orally active agent both in vitro and in vivo in colon cancer cells, while it has limited cytotoxicity in normal colonic epithelial cells [173].

#### 3.3.2 Structure‐based drug design and targeted optimization

**Fig. 3:**
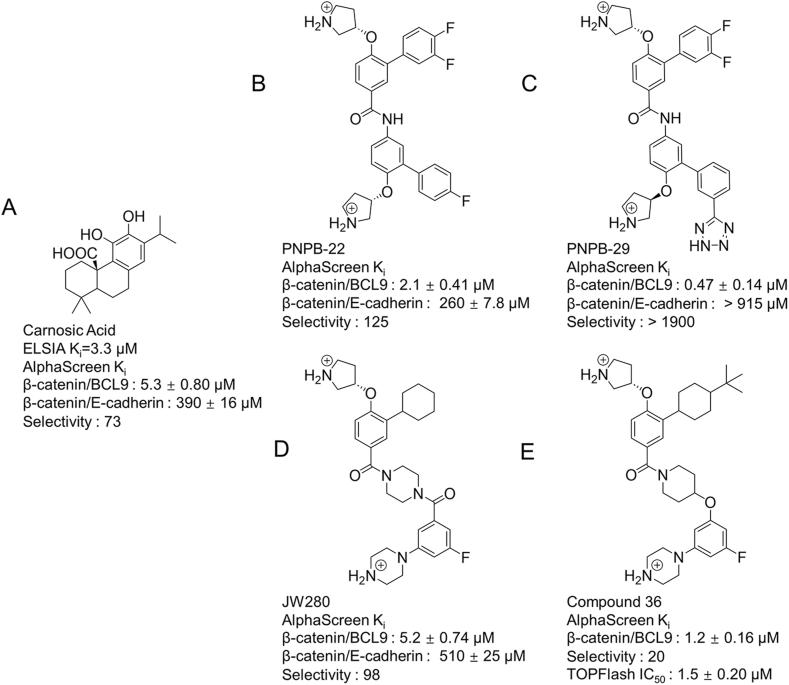
Carnosic Acid and optimization starting from PNPB-22.

The β-catenin/BCL9 protein–protein interaction (PPI) is a promising alternative target for inhibitor development of WNT/β-catenin signaling [174]. Marc et al. developed an ELISA-based ‘plus–minus' assay to identify Carnosic Acid (CA) (Fig. 3A) as a small molecule inhibitor of β-catenin/BCL9 PPI [175]. After this, small molecule inhibitors targeting the β-catenin/BCL9 PPI have been developed. Logan et al. posited that CA possesses a catechol substructure that can easily react with protein nucleophiles upon oxidation. This property may pose potential challenges in the development of inhibitors and biological research. Consequently, they designed 30 compounds for targeting the β-catenin/BCL9 PPI and verified that PNPB-22 (compound 22) (Fig. 3B) selectively disrupted the β-catenin/BCL9 PPI (AlphaScreen Ki values 2.1 ± 0.41 μM), downregulated the transcription of WNT target genes AXIN2, LGR5, LEF1, and Cyclin D1 in a dose-dependent manner. Moreover, PNPB-22 is the first class-specific small-molecule inhibitor for β-catenin/BCL9 PPIs [176]. To optimize selectivity of PNPB-22, different strategies were adopted by John and Zhang in the same research group. John et al. continued Logan's research and further designed and optimized JW280 (compound 11) (Fig. 3D) by modifying the main framework based on PNPB-22. Compound 11 can effectively inhibit the β-catenin/BCL9 interaction with 98-fold selectivity over the β-catenin/cadherin interaction [177]. However, JW280 did not show significant cell-based activities. Li et al. optimized JW280 and obtained compound 36 with excellent biological activity, as well as one of its an isomer ZL3138 after the separation of compound 36 (Fig. 3E) [178]. Zhang et al. further optimized the design of PNPB-22 by modifying the substituent groups and finally obtained a PNPB-29 (compound 29) (Fig. 3C) with higher selectivity and better potency (Ki of 0.47 μM and >1900-fold selectivity for β-catenin/BCL9 over β-catenin/E-cadherin interaction) [179].

**Fig. 4:**
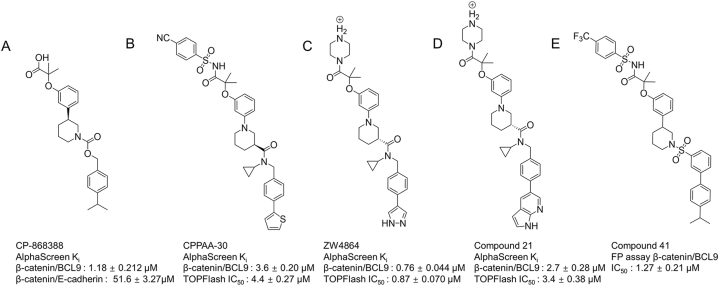
Optimization starting from CP-868388.

In another study by Zhang et al., 207 compounds were screened using AlphaScreen technology and CP-868388 was identified to exhibit a robust sigmoidal inhibitory dose response with a Ki value of 1.18 ± 0.212 μM (Fig. 4A). Although CP-868388 exhibited an excellent selection target for β-catenin/BCL9 over β-catenin/E-cadherin interactions, it is not clear whether this compound is bound to β-catenin or BCL9 [180]. Using a cell-based activity of WNT/β-catenin signaling assay [181], it had been found that CP-868388 exhibited no inhibitory activity for the transactivation of canonical WNT signaling [180]. Wang et al. performed further optimization based on CP-868388 to obtain CPPAA-30 (compound 30) (Fig. 4B) [182], ZW4864 (Fig. 4C) [183], and compound 21 (Fig. 4D) based on ZW4864 [184]. Another group also obtained another compound 41 (Fig. 4E) based on the optimization of CP-868388 [185]. These studies demonstrated that like CP-868388, these optimized compounds all exhibited excellent inhibition for β-catenin/BCL9 interaction.

**Fig. 5:**
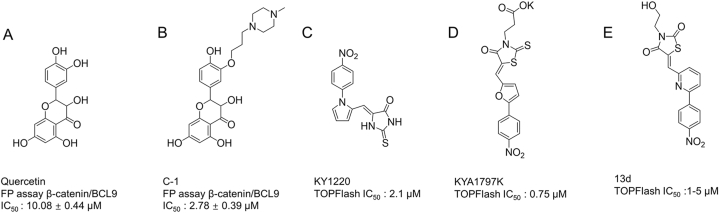
Optimization starting from Quercetin and KY1220.

Shen et al. found that Quercetin (Fig. 5A) inhibited β-catenin/BCL9 protein interaction and designed a series of compounds based on the structure of Quercetin. Further experimental screening determined that a drug-like Quercetin derivative C1 (Fig. 5B) could bind directly with β-catenin to interrupt the β-catenin/BCL9 interaction, further inhibiting hyperactive WNT signaling and proliferation and migration of CRC cells. The author also observed that C1 could regulate tumor immune microenvironment [186].

Excepting for targeting the β-catenin/BCL9 protein interaction, optimizing and designing the drugs that target other molecules of WNT signaling pathway is also a feasible approach. Cha et al. screened the small molecule compounds to obtain the lead compound KY1220 (Fig. 5C) and structurally modified it to obtain KYA1797K. They demonstrated that KYA1797K (Fig. 5D) could bind directly to AXIN, which promoted GSK3β activation to stabilize the β-catenin destruction complex, finally leading to β-catenin degradation [187]. Based on further structural modifications of KYA1797K, CHOI et al. finally identified a new compound (13d) (Fig. 5E) with stronger inhibitory potency to promote β-catenin and Ras degradation [188].

It has been reported previously that Niclosamide inhibits WNT/β-catenin signaling. To improve the selectivity of Niclosamide for WNT signaling and its pharmacokinetic properties, Robert et al. designed and synthesized a series of Niclosamide derivatives based on SAR studies of the Niclosamide salicylanilide chemotype. They identified a target compound with greater selectivity for WNT/β-catenin signaling inhibition and less effect on cellular ATP homeostasis [189]. Based on a triazole motif, further studies also identified a new structural class of WNT/β-catenin signaling inhibitors that could induce the internalization of FZD-1 receptors and inhibit the transcription activity of WNT/β-catenin signaling [190]. To improve the diuretic properties and relative lack of potency of ethacrynic acid (EA), Jin et al. designed and synthesized 40 amide derivatives of EA, and finally obtained several target compounds with enhanced WNT inhibitory effect and potency in inhibiting the growth of chronic lymphocytic leukemia cells [191].

### 3.4 Design of the peptide-based inhibitors inactivating the WNT signaling pathway

Peptide drugs have been widely used in clinical practice and have many advantages with high specificity, good efficacy, good safety, membrane permeability [192]. Recently, peptide-based inhibitors have been attracting attention increasingly and become an alternative therapy for targeting the WNT signaling pathway. Here, we focus on some peptides-based inhibitors of WNT signaling pathway (Table 3). Based on the transmembrane domain of Klotho protein, Chen et al. designed a short peptide KP6 with 30 amino acids (QPVVTLYHWDLPQRLQD-AYGGWANRALADH) and a WNT-binding motif, which binds competitively with WNT to block the activation of WNT signaling pathway [193]. Xie et al. reported a recombinant soluble peptide fragment (rhFzd7) that interrupts the binding of Fzd7 with WNT ligand. rhFzd7 exhibited a high affinity with WNT3a (K_d_ = 3.41 × 10^−8^ M), and induced effectively apoptosis of MDA-MB-231 cells [194]. The peptide Fz7-21 (LPSDDLEFWCHVMY) has been also reported to have ability to inhibit the WNT signaling pathway probably through disrupting the formation of WNT/FZD/LRP complex [195].

**Table 3:** Peptide-based blockers with WNT inhibitory activity.

Name	Sequence	Function Mechanism
KP6	QPVVTLYHWDLPQRLQDAYGGWANRALADH	Binding to WNT ligands and disrupting the engagement of WNTs with receptor-related protein 6 [193]
rhFzd7	/	Binding of the FZD7 extracellular CRD with WNT ligands competitively and further inhibiting the functional activity of FZD7 [194]
Fz7-21	LPSDDLEFWCHVMY	Disrupting the formation of a WNT3A–FZD7–LRP6 ternary complex, leading to inhibition of Wnt signaling [195]
ACBP	/	inhibiting phosphorylation of LRP6, further leading to downregulation of WNT target genes [196]
P^182−195^	MEENAYQVFLTSDI	Activating of endogenous AXIN2 to increase the specificity for cancer cells and reducing Wnt/β-catenin signaling [197]
StAx-35/StAx-35R/NLS-StAx-h	/	Targeting and binding with β-catenin directly, inhibiting the interaction between β-catenin and corresponding transcription factors [198,199]

β-catenin is the key regulator of WNT signaling pathway and its stability plays an important role in WNT/β-catenin signaling pathway. Some peptide regulators can regulate the WNT/β-catenin signaling pathway by controlling β-catenin levels. Anticancer bioactive peptide (ACBP) was isolated from spleens of goats immunized with tumor extracts. ACBP was demonstrated to promote β-catenin phosphorylation and degradation mainly through inhibiting phosphorylation of LRP6, further leading to downregulation of WNT target genes such as cyclin D1, met, c-Myc [196]. Conductin is a key factor in the negative regulation of β-catenin and its polymerization shows high activity for suppressing WNT signaling [200]. Bernkopf et al. design a short peptide P^182−195^ (MEENAYQVFLTSDI) that induces conductin polymerization and promotes β-catenin degradation [197]. To antagonize the β-catenin/TCF interaction, Grossmann et al. designed two hydrocarbon-stapled peptides (StAx-35 and StAx-35R) that can bind directly with β-catenin and suppress WNT target gene expression such as LEF1, LGR5 [198]. To optimize protein stability and maximize cell uptake, the nuclear localization sequence (NLS) was introduced to the stapled peptide StAx-35R and all R residues of the peptide StAx-35R were replaced with homoarginine, named NLS-StAx-h [199]. Similarly, NLS-StAx-h effectively disrupted the interaction of β-catenin with TCF and suppressed WNT target gene expression, resulting in inhibition of proliferation (IC_50_ 1.4 μM) and migration of CRC cells.

Except for the traditional peptide blockers, researchers also design peptides that have the capability to inhibit PPI by mimicking protein secondary structure motifs in silico. Using the Rosetta suite of protein design algorithms, Schneider et al. reported that a serial of macrocycles composed of peptide ubunits could target the β-catenin/TCF interaction. By an in vitro luciferase reporter system and a zebrafish model, the most active macrocycle was identified to have the ability of the proliferation of prostate cancer cell lines through inhibiting both WNT and AR-signaling [201].

Although peptide-based inhibitors of the WNT/β-catenin signaling pathway have shown promising clinical potential and provide a new direction for future cancer therapies, the safety and efficacy of these peptide-based inhibitors will be considered essentially. A comprehensive understanding of the characteristics of peptide-based inhibitors such as physical and chemical properties, clearance rate, delivery and so on, will be worthy of further study for extending the scope of clinical application.

## 4 Immunotherapy

**Fig. 6:**
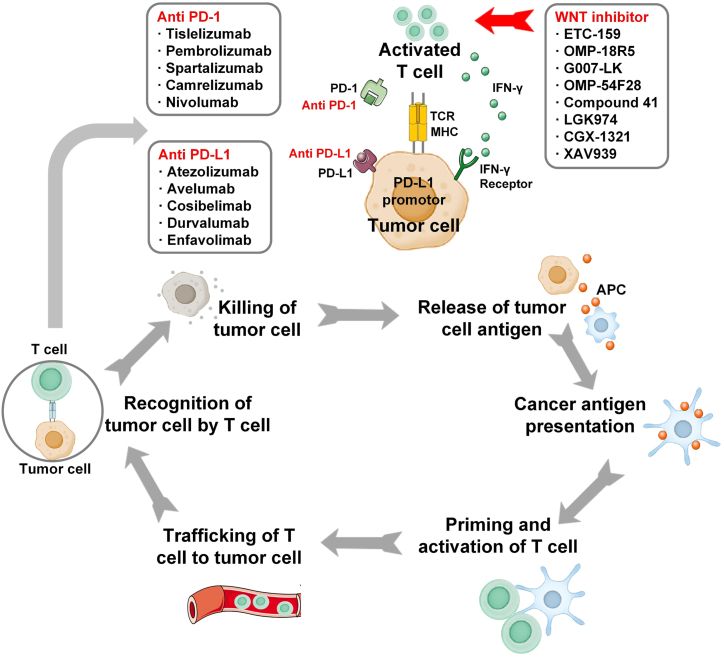
Immune response against cancer and WNT inhibitors. APC, antigen-presenting cell; TCR, T cell receptor; MHC, major histocompatibility complex; PD-1, programmed cell death protein-1; PD-L1, programmed cell death protein ligand 1.

Immunotherapy is a therapy that impedes the suppression of immune effects and restores or enhances the existing immune response against cancer [202]. The anti-cancer immune response requires the initiation of a series of events. First, the antigens produced by cancer cells are captured by dendritic cells (DCs). DCs then present the antigens captured on MHC I and MHC II molecules to T and B cells to activate effector T cells for cancer-specific antigens, where the ratio of effector T cells to regulatory T cells is critical for the outcome. Finally, activated effector T cells infiltrate the tumor site by interacting with the T cell receptor (TCR), and its cognate antigen bound to MHC I specifically recognizes and binds to the cancer cells to kill the cancer cells. The killed cancer cells release additional tumor-associated antigens which result in cascade amplification of this process [203,204]. However, molecular factors constituting the intracellular interferon pathway are defective in cancer cells, resulting in cancer cells evading immune surveillance [205]. This also provides a new strategy for immunotherapy of cancer, thus triggering a major success in immunotherapy. Immune checkpoint inhibitors (ICIs) have now entered medical practice and become one of the most important immunotherapies, with breakthroughs in many cancer types [206]. However, ICIs are also clinically resistant, and studies have now identified WNT/β-catenin pathway activation as a major cause of resistance to ICIs [207]. For example, β-catenin signaling in melanoma leads to T-cell rejection and resistance to anti-PD-L1 and anti-CTLA-4 monoclonal antibody therapy [208]. A bioinformatics study found reduced CD8^+^ T-cell infiltration in colon cancer with high β-catenin expression, indicating a promising application of targeting the WNT/β-catenin signaling pathway combined with immune checkpoint blockade for the treatment of CRC [209]. DeVito also indicated that WNT inhibitors (OMP-18R5, OMP-54F28, ETC-159) hold promise for reversing tumor-mediated immune tolerance and enhancing the efficacy of PD-1 blockade [210]. Thus, the combined application of WNT inhibitors may be a powerful modality to improve cancer efficacy during cancer immunotherapy (Fig. 6).

On the one hand, the application of WNT inhibitors can improve the therapeutic effect of immunotherapy (Table 4). Waaler et al. found that inhibition of the WNT pathway using a tankyrase inhibitor G007-LK in a mouse model of melanoma could sensitize the tumors to anti-PD-1 immune checkpoint therapy. Meanwhile, G007-LK also induced IFN-γ and CD8^+^ T cell-dependent anti-tumor immune responses [211]. Another study by DeVito et al. also showed that the combination of WNT inhibitors with aPD1 enhanced the immunotherapeutic effect by inhibiting WNT signaling. This combination treatment could convert the tumor microenvironment to a more favorable state to generate an effective anti-tumor immune response to aPD1 in melanoma and NSCLC cells [210]. It has been reported that a novel WNT inhibitor synthesized by Zhang et al. could enhance the infiltration and function of cytotoxic T lymphocytes (CTLs) and inhibit the infiltration of regulatory T-cells (Tregs). The combination of this novel WNT inhibitor with an anti-PD-1 antibody suppressed resistance to PD-1 therapy in a CT26 tumor model, thereby enhancing the efficacy against CRC in vivo [185].

**Table 4:** WNT signaling blockers with clinical process in immunotherapy.

Compounds	Indications	Process
**OMP-18R5** [210]	pancreatic cancer;	Phase Ⅰ Clinical Trial
	Metastatic breast cancer	
	Solid tumors	
**OMP-54F28** [210]	Ovarian Cancer	Phase Ⅰ Clinical Trial
	Pancreatic Cancer	
	Solid Tumors	
**ETC-159** [210]	Advanced Solid Tumors	Phase Ⅰ Clinical Trial
**G007-LK** [211]	N/A	Preclinical Stage
**LGK974** [212]	Metastatic Colorectal Cancer	Phase Ⅱ Clinical Trial
	Solid Tumors	
**DKN-01** [213]	Endometrial Cancer	Phase Ⅱ Clinical Trial
	Colorectal Cancer;	
	Gastric Cancer	
	Metastatic Esophageal Cancer	
	Prostate Cancer	
**CGX-1321** [213]	Advanced Gastrointestinal Tumor	Preclinical Stage

On the other hand, the application of WNT inhibitors for tumors that are not sensitive to immunotherapy can enhance the sensitivity of cancer cells to immunotherapy. For example, in pancreatic ductal adenocarcinoma, PD-L1 blockade therapy is usually ineffective, but the addition of the WNT inhibitor LGK974 allows PD-L1 blockade therapy to exert an inhibitory effect on the growth of pancreatic cancer. The mechanism is due to changes in the immune microenvironment caused by WNT signaling inhibition and increased CD8^+^ T cell-mediated immune response, making pancreatic cancer sensitive to PD-L1 blockade therapy [212]. It has been reported that PD-1 ICIs are ineffective in the treatment of glioblastoma due to insufficient immune infiltration. A study from Zhang et al. suggested that direct inhibition of tumor proliferation and migration by blocking WNT/β-catenin signaling could enhance the efficacy of PD-1 blockade therapy in glioblastoma, along with the promotion of T-cell infiltration and PD-L1 expression in the tumor microenvironment [214]. Li et al. also reported that inhibition of the WNT pathway could improve the sensitivity of gastric cancer cells to PD-1 blocking therapy [215].

In a study of ovarian cancer, the addition of DKK1 inhibitor DKN-01 or anti-PD-1 therapy did not enhance the antitumor effect of PORCN inhibitor CGX-1321, although the use of CGX-1321 monotherapy significantly reduced tumor burden and increased CD8^+^ T-cell levels [213]. The same group attempted to change the strategy that using DKK1 monoclonal antibody to activate the WNT pathway. As expected, DKN-01 monotherapy could increase the expression of WNT target genes and promote MHC I regulators expression in a mouse model. The use of WNT inhibitor CGX-1321 further increased the production of T-cell chemokine CXCL10, demonstrating that sequential regulation of the WNT/β-catenin signaling pathway may sensitize tumors to immunotherapy for improving tumor immune evasion [216].

Despite targeting the WNT signaling pathway plays roles in improving tumor immunotherapy, WNT signaling in immunotherapy should not be blindly blocked. Inhibition of WNT signaling in some tumors may be detrimental to ICI therapy and may reduce the antigenicity of the tumor, thereby compromising immunotherapy efficacy. Therefore, the roles of WNT signaling pathway in immunotherapy still need to be further studied for improving the efficiency of immunotherapy, which will benefit the application of drugs targeting WNT signaling in anti-tumor immunity.

## 5 Gene therapy targeting the WNT signaling pathway

It is well known that gene therapy exerts its effects by transferring genetic material (nucleic acids, viruses, or genetically engineered microorganisms) into the cells. The concept of gene therapy was first put forward nearly fifty years ago for the treatment of inherited monogenic disorder. Nowadays, gene therapy has been applied to various diseases such as cancer [217].

**Fig. 7:**
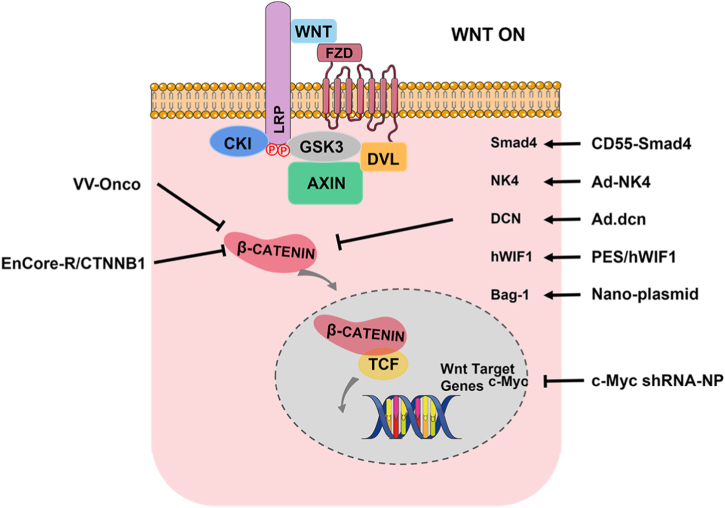
Targeting of WNT signaling pathway gene therapy strategies.

Cancer gene therapy is divided into two categories: directly targeting tumor cells or host cells to suppress tumor growth [218]; using effective gene delivery systems that allow the safe transfer of genes to target tissues, bypassing the immune system [219]. Given the importance of the WNT signaling pathway in tumor occurrence, development, and metastasis, several gene therapy systems targeting the WNT signaling pathway have been developed and exert good effects in anticancer therapy (Fig. 7).

### 5.1 Virus as a carriers

Smad4 is a key signal transduction protein of the transforming growth factor-β (TGF-β) pathway. In many cancers, high expression of Smad4 could suppress tumor growth and metastasis, and loss of function of Smad4 promotes tumor progression. Based on a CEA-controlled oncolytic adenovirus (CD55), Xiao et al. constructed a novel oncolytic adenovirus that harbors the Smad4 gene, named CD55-Smad4, and tested the potent antitumor efficacy of CD55-Smad4 in CRC. Experimental results showed that CD55-Smad4 could suppress the migration, invasion, and stemness of CRC cells by inhibiting the WNT/β-catenin signaling pathway, thus exerting anti-cancer effects [220].

Jia et al. constructed a thymidine kinase gene insertional inactivated Vaccinia virus (VV-Onco) and found that VV-Onco could induce apoptosis and inhibit the colony formation of MHCC97-H cells, along with the downregulation of Survivin and c-Myc, which is attributed to the inhibition on endoplasmic reticulum stress, autophagy and WNT signaling pathway [221]. However, future studies still need to detect the anti-tumor efficacy of VV-Onco in an in vivo model.

NK4 is a hepatocyte growth factor (HGF) antagonist and angiogenesis bifunctional inhibitor. The NK4 adenovirus constructed by Deng et al. was demonstrated to have the ability to inhibit cell proliferation, invasion, and tumorigenesis through impeding Met/AKT/β-catenin signaling in human malignant mesothelioma. Mechanistically, NK4 expression could inhibit β-catenin nuclear localization to regulate its transcriptional activity, further resulting in the downregulation of downstream target genes Oct4 and c-Myc, ultimately exerting a suppressive effect on tumor stem cells [222].

Decorin, a small leucine-rich proteoglycan, is considered an attractive candidate to inhibit prostate cancer tumorigenesis and bone metastases since decorin protein has been demonstrated to target multiple signaling pathways associated with prostate cancer tumorigenesis and bone metastases, including the WNT/β-catenin signaling pathway. Therefore, Xu et al. constructed a recombinant oncolytic adenovirus Ad-dcn carrying the human decorin gene, which could significantly reduce the expression of β-catenin, VEGFA and result in the inhibition of prostate cancer bone metastases [223].

### 5.2 Liposomal nanoparticle carriers

Mao found that lncRNA-SLERCC (SLERCC) was lowly expressed in renal cell carcinoma, and further exploration revealed that SLERCC interacted directly with UPF1 and inhibited renal cell carcinoma development through the WNT/β-catenin signaling pathway. Based on this, the Plasmid-SLERCC@PDA@MUC12 nanoparticles (PSPM-NPs) were constructed to overcome the lncRNA instability using the encoding plasmid and improve the target of SLERCC to RCC cell lines by the incorporation of transmembrane metastasis marker MUC12 antibody. The results demonstrated that the PSPM-NPs nanotherapeutic system could effectively renal cell carcinoma progression and metastasis [224].

RNA interference (RNAi) is often applied to previously-undruggable targets. β-catenin is a central critical factor of the WNT signaling pathway, and there is no approved therapy targeting β-catenin. Ganesh et al. developed a lipid nanoparticle that can deliver siRNA targeting *CTNNB1* to tumor tissue and generate mRNA silencing and antitumor efficacy in multiple mouse modes. Notably, tumor growth was significantly inhibited after EnCore-R/*CTNNB1* treatment in WNT-driven colorectal and HCC models [225].

### 5.3 Polymer nanoparticle carriers

In CRC, both WNT/β-catenin and RAS-ERK pathways are often aberrantly activated because of the high frequency of adenomatous polyposis coli (APC) and KRAS mutations. Based on CRISPR-Cas9 technology, Wan et al. designed and synthesized a hyaluronic acid (HA)-decorated phenylboronic dendrimer (HAPD) to target APC and KRAS mutations by delivering Cas9 ribonucleoprotein (RNP) for the treatment of CRC. As expected, HAPD significantly inhibited CRC tumorigenicity and reduced CRC liver metastasis and lung metastasis, indicating that these duplex genome-editing nanoparticles have a potential application value for the treatment of tumors carrying APC and KRAS mutations [226].

Among non-viral vectors, cationic polyethyleneimine (PEI) as efficient gene vectors have been studied extensively. WIF-1, a secreted protein, is known as a WNT antagonist that can bind WNT ligands. Based on short PEI chains and the target peptide SP5-2, PEI-SP5-2 (PES) was synthesized and used as a gene delivery vector for the in vivo delivery of the human WIF-1 gene. The newly synthesized particles PES/hWIF-1 was demonstrated to have potency for growth inhibition by blocking the WNT signaling pathway [227]. In another study, Tangudu's group used the macromolecule polyglycol methacrylate (PGMA) conjugated with multiple PEI chains as a platform to deliver c-Myc shRNA. The obtained nanoparticles loading c-Myc shRNA exhibited an efficient inhibition in both breast tumors bearing mutant BRCA2/p53 and APC-deficient CRC transgenic mouse [228].

Although these nanoparticle delivery systems show promising application prospects, they are rarely adopted in practical clinical applications because of their high cost, unpredictable side reactions, the complicated preparation process, and involved social ethical issues et al. Many new delivery systems are still under further development. Ishiguro et al. used extracellular vesicles isolated from milk loaded with β-catenin siRNA to develop an engineered biological nanoparticle. To improve the target of nanoparticles, RNA aptamers capable of binding to EpCAM were incorporated into the decorated RNA scaffolds. EpCAM is a target of WNT/β‐catenin signaling and a marker of CSCs. By targeting EpCAM-expressing liver CSCs, the nanoparticles have been experimentally demonstrated to have anti-cancer effects and inhibit the WNT signaling pathway when used to deliver β-catenin siRNA [229]. Huang et al. used magnetic gold nanoparticles for loading with the silencing Bag-1 gene plasmid pGPH1/GFP/Neo-Bag-1-homo-825 and found it to be effective against CRC both in vivo and in vitro, which may be related to the inhibition of WNT/β-catenin signaling pathway [230].

MicroRNAs (miRNAs) are small noncoding RNAs with 18–25 nucleotides long that manipulate precisely gene expression. It has been reported that many miRNAs can target β-catenin. Using a dual-fluorescence FunREG screening system, Indersie et al. screened a library of 1712 miRNAs mimics and identified four new miRNAs that could decrease the expression and transcriptional activity of β-catenin. Interestingly, miR-624-5p directly targeted the 3′-UTR of the three β-catenin mRNA variants and reduced the oncogenic function of the WNT/β-catenin signaling pathway, leading to tumor growth inhibition, suggesting that miR-624-5p may be a promising target for gene therapy [231].

The precise targeting of the WNT pathway by gene therapy has also made it possible to combine gene therapy with chemotherapy. Based on a combined chemotherapy and gene therapy treatment strategy, Lo et al. constructed two nanoparticles, omSLN-CMN and omLip-CMN. Both nanoparticles are wrapped using pH-responsive PEG with a lipid core consisting of N peptide for tumor targeting, M peptide for mitochondrial targeting, and C peptide for enhanced cancer penetration, and then incorporated with miR-200 and Irinotecan, respectively. Experimental results showed that treatment with combinatorial therapy induced cell apoptosis by regulating the WNT/β-catenin signaling pathway and EMT pathways [232].

## 6 Physical therapy

In addition to direct physical therapy such as surgical removal of tumor tissue, other physical therapies for cancer treatment such as radiation therapy (RT) and photodynamic therapy (PDT) are also flourishing. The presence of WNT signaling activation can also affect the efficacy of these physical treatments.

### 6.1 Radiation therapy

RT, as one of the main tools in cancer treatment, has been widely used in various malignant tumors [233]. The treatment can damage cancer cells by causing DNA damage either directly or indirectly through the production of reactive oxygen species (ROS) [234]. Paradoxically, RT including ionizing radiation (IR) has been reported to promote tumor recurrence and metastasis [233,[235], [236], [237]]. Therefore, successful searching for an effective way to avoid tumor recurrence and metastasis will be an important improvement for RT treatment of tumors, especially those that cause patients severe discomfort, resist other treatments, or cannot be removed surgically.

It has been reported that the WNT signaling pathway plays a role in radiotherapy resistance [[238], [239], [240]]. Lu et al. found that radiation significantly promoted the expression of the WNT pathway ligand in nasopharyngeal carcinoma, especially WNT5A, a typical ligand of the WNT/β-catenin signaling pathway. The experimental results proved that WNT5A expression could reduce the radiosensitivity of nasopharyngeal carcinoma, and knockdown of Beclin1 which is a main target of WNT5A partially reduced WNT5A-mediated protective autophagy in promoting radiation resistance [241]. The study of Li et al. showed that WNT5a knockdown combined with radiotherapy could inhibit proliferation and induce apoptosis of NSCLC cells, and these effects were reversed following WNT5a overexpression or β-catenin knockdown [242]. CircRNA ZNF292 (CZNF292) was also found to reduce the activity of the WNT/β-catenin pathway and inhibit the proliferation and radio resistance of hypoxic HCC cells in vitro and in vivo [243]. Therefore, inhibition of the WNT pathway combined with RT may provide assistance in sensitizing RT and attenuating RT resistance.

Yin et al. found that Niclosamide, a potent inhibitor of WNT/β-catenin signaling, not only inhibited intrinsic WNT/β-catenin signaling, but also blocked IR-induced WNT/β-catenin signaling [244]. Except for enhancing the sensitivity of tumor cells to IR, Niclosamide also blocked WNT3a-induced radio resistance and overcame β-catenin-induced radio resistance, providing experimental evidence for the combination of Niclosamide with RT for patients. In addition, it has been shown that doxycycline, a DNA-PK inhibitor, can inhibit multiple pathways such as WNT, Sonic Hedgehog, Notch, and TGF-β signaling, which can sensitize tumor cells to RT, indicating that the application of doxycycline can improve the tumoricidal efficiency of RT [245]. The use of the WNT inhibitor LGK-974 similarly increased the sensitivity of HCC cells to radiotherapy [246]. Together, these results suggest that the WNT/β-catenin signaling pathway plays an important role in the development of tumor cell radio resistance. In most cancers, aberrant activation of the WNT/β-catenin signaling pathway is often observed. Therefore, in clinical radiotherapy, the application of WNT inhibitors or suppression of the activation of the WNT signaling pathway by other ways such as gene editing may effectively attenuate RT resistance and provide a valuable strategy for RT.

### 6.2 Photodynamic therapy (PDT)

PDT is one of the promising treatments currently used for tumor treatment. The principle of PDT is to use the selective aggregation property of photosensitizers to kill tumor cells [247,248]. The concentration of photosensitizers in tumor tissue is significantly higher compared to normal tissue [249]. The photosensitizer itself is non-toxic unless activated by a specific wavelength of light [250]. Although the mechanism of action of PDT is not fully understood, a large body of clinical evidences suggest that PDT is an effective treatment option for some specific types of tumors [251]. It has been found that WNT signaling and cancer cell proliferation may be inhibited during PDT. Han et al. found that PDT could induce p53-mediated miR-34a-5p expression in an Ago2-dependent manner, and miR-34a-5p could target WNT7B and inhibit expression of WNT7B, resulting in the inactivation of WNT signaling [252]. It is the inhibition of the WNT signaling pathway by PDT that enhances the sensitivity of cancer cells to PDT and improves the efficacy of the treatment. This finding is of great value for PDT treatment of cancer and related studies on the WNT signaling pathway. A related study reported by Zuo et al. also found that in pyromellitic chlorophyll-α-methyl ester-mediated PDT (MPP-α-PDT), silencing GRP78 expression could inhibit the WNT signaling pathway and improve the sensitivity of HOS osteosarcoma cells to MPP-α-PDT, thereby improving the efficacy of cancer treatment [253].

The clinical application of PDT is limited by the lack of light penetration depth, the low bioavailability of photosensitizers, the low oxygen content in tumors, repetitive treatment, and so on [254]. The recurrence rate of tumors is also relatively high after PDT. It is well known that the WNT signaling pathway plays an important role in tumor recurrence and metastasis [255]. At present, there are relatively few studies on the relationship between PDT and WNT signaling pathway. Therefore, it is of great significance to study the changes in the WNT signaling pathway during PDT and the roles of the WNT signaling in PDT treatment for improving the clinical therapeutic effect of PDT, which will provide a new therapeutic strategy for tumor treatment.

### 6.3 Other physiotherapeutic

Except for RT and PDT, the emergence of other forms of physical therapy such as photothermal therapy (PTT), sonodynamic therapy (SDT), microwave ablation (MA), and so on, provides more potential for improving the efficiency of cancer treatment. PTT is a type of therapy that uses materials with the efficiency of photothermal conversion to kill cancer cells. Photothermal materials are gathered around tumor tissues with targeted recognition technology and can convert light energy into heat energy under external light (usually near-infrared (NIR) light). Similar to PDT, PTT is often used in patients with superficial tumors [[256], [257], [258], [259]]. For anticancer studies under PTT, it is generally believed that photothermal agents can be released in the tumor or lesion area. When illuminated with NIR light, it produces high temperatures (>46 °C) that can effectively kill tumor cells. Zhang et al. designed porous AuPd nanoparticles as NIR photothermal agents to generate mild local heat (MLH) (40-43 °C) via PTT. RNA-seq analysis showed that MLH could lead to the expression of osteocalcin and promote bone regeneration through upregulating the expression of WNT signaling activators WNT10b and A1p1, suggesting that the WNT signaling pathway may be involved in MLH [260]. However, this conclusion lacks experimental verification, and further study is essential for dissecting the effects of WNT signaling during MLH. In addition, under PTT-mediated high temperature (>46 °C), whether it is associated with the anti-cancer research of the WNT pathway may become a new research topic, and its anti-cancer effect needs to be further studied and verified. The use of some new methods combined with the inhibition of WNT-related pathways may become a promising research hotspot in the future.

## 7 Discussion

The WNT pathway acts as a double-edged sword throughout biological growth and development and is extensively involved in cancer development and progression. How to properly and precisely control the WNT pathway to fight cancer is still a question that needs to be carefully studied. With the in-depth study of the WNT pathway, it has been found that the WNT pathway is a great obstacle to cancer treatment, which should draw more attention to tumor therapy. In the existing cancer treatment modalities, it will be of important clinical significance to successfully solve the problem of how the existing treatment synergizes effectively with targeting the WNT pathway. Therefore, we first must clarify the roles of the WNT signaling pathway including various signaling components in the different therapeutic modalities. Secondly, it is urgently necessary to find and develop new targeted drugs based on the existing natural compounds or clinical drugs using structural modification or/and conventional drugs in new use, since there are no available drugs targeting the WNT signaling pathway in clinical cancer therapy.

Here, we review a large number of literatures to describe the development of WNT inhibition and hope to provide research ideas to find more efficient and specific WNT pathway inhibition. Subsequently, we summarize some research results from the studies about direct inhibition of the WNT pathway or combination of WNT pathway inhibitors with various therapies, suggesting that the combination therapy can get more expected therapeutic results compared with the monotherapy such as chemotherapy, immunotherapy, and gene therapy. This inspires us that future research in cancer treatment should not only directly kill cancer cells but also target the WNT pathway to improve tumor sensitivity to different modalities and enhance efficacy, even long-term efficacy.

## Ethical approval

Not applicable.

## Compliance with ethical standards

Not applicable.

## Consent to participate

Not applicable.

## Consent to publish

Not applicable.

## Funding

This study is supported by Guangxi Natural Science Foundation (2024GXNSFAA010027, 2024GXNSFAA010456, 2020GXNSFBA297123), 10.13039/501100001809National Natural Science Foundation of China (U21A20421, 82360719), Young and middle-aged teachers research basic ability improvement project of GuangXi (2021KY0122), Innovation Project of Guangxi 10.13039/100000082Graduate Education (YCSW2023014).

## Data availability statement

All the data obtained during this study are included in the manuscript. No data was used for the research described in the article.

## CRediT authorship contribution statement

**Xi Zhao:** Writing – review & editing, Writing – original draft, Visualization, Validation, Supervision, Software, Resources, Project administration, Methodology, Investigation, Funding acquisition, Formal analysis, Data curation, Conceptualization. **Yunong Ma:** Writing – review & editing, Writing – original draft, Visualization, Validation, Supervision, Software, Resources, Project administration, Methodology, Investigation, Funding acquisition, Formal analysis, Data curation, Conceptualization. **Jiayang Luo:** Software. **Kexin Xu:** Validation, Supervision. **Peilin Tian:** Software, Methodology. **Cuixia Lu:** Writing – review & editing. **Jiaxing Song:** Writing – review & editing, Writing – original draft, Visualization, Validation, Supervision, Software, Resources, Project administration, Methodology, Investigation, Funding acquisition, Formal analysis, Data curation, Conceptualization.

## Declaration of competing interest

The authors declare that they have no known competing financial interests or personal relationships that could have appeared to influence the work reported in this paper.
